# Epigenomic and transcriptomic persistence of heat stress memory in strawberry (*Fragaria vesca*)

**DOI:** 10.1186/s12870-024-05093-6

**Published:** 2024-05-16

**Authors:** María-Estefanía López, Béatrice Denoyes, Etienne Bucher

**Affiliations:** 1 Crop Genome Dynamics Group, Agroscope, Nyon, 1260 Switzerland; 2 Department of Botany and Plant Biology, Faculty of Sciences, University of Geneva, Geneva, 1205 Switzerland; 3 INRAE, Biologie du Fruit et Pathologie, Univ. Bordeaux, Villenave d’Ornon, F-33140 France

**Keywords:** Fragaria, Epigenetic, Memory, Stress, Asexual, Reproduction

## Abstract

**Background:**

In plants, epigenetic stress memory has so far been found to be largely transient. Here, we wanted to assess the heritability of heat stress-induced epigenetic and transcriptomic changes following woodland strawberry (*Fragaria vesca*) reproduction. Strawberry is an ideal model to study epigenetic inheritance because it presents two modes of reproduction: sexual (self-pollinated plants) and asexual (clonally propagated plants named daughter plants). Taking advantage of this model, we investigated whether heat stress-induced DNA methylation changes can be transmitted via asexual reproduction.

**Results:**

Our genome-wide study provides evidence for stress memory acquisition and maintenance in *F. vesca*. We found that specific DNA methylation marks or epimutations are stably transmitted over at least three asexual generations. Some of the epimutations were associated with transcriptional changes after heat stress.

**Conclusion:**

Our findings show that the strawberry methylome and transcriptome respond with a high level of flexibility to heat stress. Notably, independent plants acquired the same epimutations and those were inherited by their asexual progenies. Overall, the asexual progenies can retain some information in the genome of past stresses encountered by their progenitors. This molecular memory, also documented at the transcriptional level, might be involved in functional plasticity and stress adaptation. Finally, these findings may contribute to novel breeding approaches for climate-ready plants.

**Supplementary Information:**

The online version contains supplementary material available at 10.1186/s12870-024-05093-6.

## Background

In order to adapt to global climate change, plants need to acquire heritable variations at the genetic and epigenetic levels [1]. For instance, modification of floral transition is one of the life strategies plants can deploy to adapt to changing environments [2]. In contrast to a single flowering event in annual plants, perennials (plants that live more than two years) can flower and restart vegetative growth every year [3, 4]. Many perennial herbaceous plants have two types of reproduction: sexual and asexual (vegetative). These include plants such as cassava (*Manihot esculenta*), greasewood (*Larrea tridentata*), blackbrush (*Coleogyne ramosissima*), and woodland strawberry (*Fragaria vesca*) [5]. Plants that reproduce *via* asexual reproduction generate genetically identical ramets or daughter plants from different tissues such as roots, rhizomes, stems (including stolons, elongated stems, and tubers, modified stems), leaves, and inflorescences [6, 7]. Compared to sexual reproduction, asexual reproduction results in reduced genetic diversity, leading to a gradual loss of adaptative capacity [7]. This could be attributed to the short-distance dispersal strategy of plants, resulting in a limited ability to avoid adverse environmental conditions [8]. However, asexually propagated plants can differ from the parental plant due to the accumulation of random genetic mutations (somaclonal variation) contributing to phenotypic variability [9, 10].

In an asexual population, aside from somaclonal genetic variations, epigenetic changes can also impact the function of the genome, thereby contributing to heritable phenotypic variation [11]. The extension of lateral meristems from stolons (St) in *F. vesca* can produce lateral buds to generate complete daughter plants [12]. It was demonstrated that asexual daughter plants of *F. vesca* grown in soil exhibit low genetic mutation rates, approximately 0.6 mutations per plant. However, in vitro tissue culture, alterations in DNA methylation levels increase both somaclonal genetic and epigenetic variation frequency [12–14]. Previous studies have investigated DNA methylation levels associated with the climate of origin, including factors such as temperature and altitude, in natural plant populations, including *F. vesca* [15–19]. Likewise, our latest work showed how dynamic the epigenome of *F. vesca* can be at the DNA methylation level when plants are subjected to a diverse range of stress conditions [20]. The consistent transmission of these epigenetic marks or epimutations may play a crucial role in shaping the adaptation and biological diversity observed in both asexual and sexual generations. For example, exposure to phosphate starvation and drought stress in rice cultivars, induced short-term memory in newly grown leaves, characterized by altered DNA methylation levels associated with stress response genes [21, 22]. Similarly, in clonal white clover (*Trifolium repens*), consecutive exposures to drought stimulated methylation changes that persisted across multiple asexual generations [23]. For Arabidopsis, it has been shown that hyperosmotic stress-induced gain and loss of DNA methylation can be heritable to subsequent generations through the maternal line [24]. In our previous work, we demonstrated that heat stress in *F. vesca* was particularly interesting from an epigenetic perspective because it led to the most pronounced genome-wide and local loss of DNA methylation among all the tested stresses [20]. In this study, we aimed to investigate the impact of heat stress on DNA methylation and gene expression and explore the potential transmission of this information to subsequent asexual generations. In summary, we observed stable inheritance of DNA methylation changes at cytosines in symmetric sequence contexts (CG and CHG) throughout asexual reproduction. Conversely, the original DNA methylation states, particularly in the CHH context, were largely reinstated in the second asexual reproduction cycle. Nevertheless, specific epigenetic changes persisted across multiple generations, indicating that heat stress has the potential to induce enduring epimutations.

## Materials and methods

### Plant material and growing conditions

We used the homozygous near-isogenic line (NIL, Fb2:39–47), *F. vesca cv. Reine des Vallées* (RV) [20, 25]. This line is everbearing as it carries the R locus allowing it to develop runners in long day conditions (LD) [25]. Sterilized seeds were germinated in water on Whatman filter paper for two weeks and transferred to 1/2 Murashige & Skoog (MS) medium (Duschefa cat# M0222), 30% sucrose, and 2% Phytagel (Sigma‒Aldrich cat# P8169). Plants were grown for four weeks under long-day control conditions (16 h light 24 °C/8 h dark 21 °C; 150 µmol m^− 2^ s^− 1^) in growth chambers (Panasonic, phcbi: MLR-352/MLR-352 H) prior to stress.

### Stress assays in vitro for mother plants

**Fig. 1:**
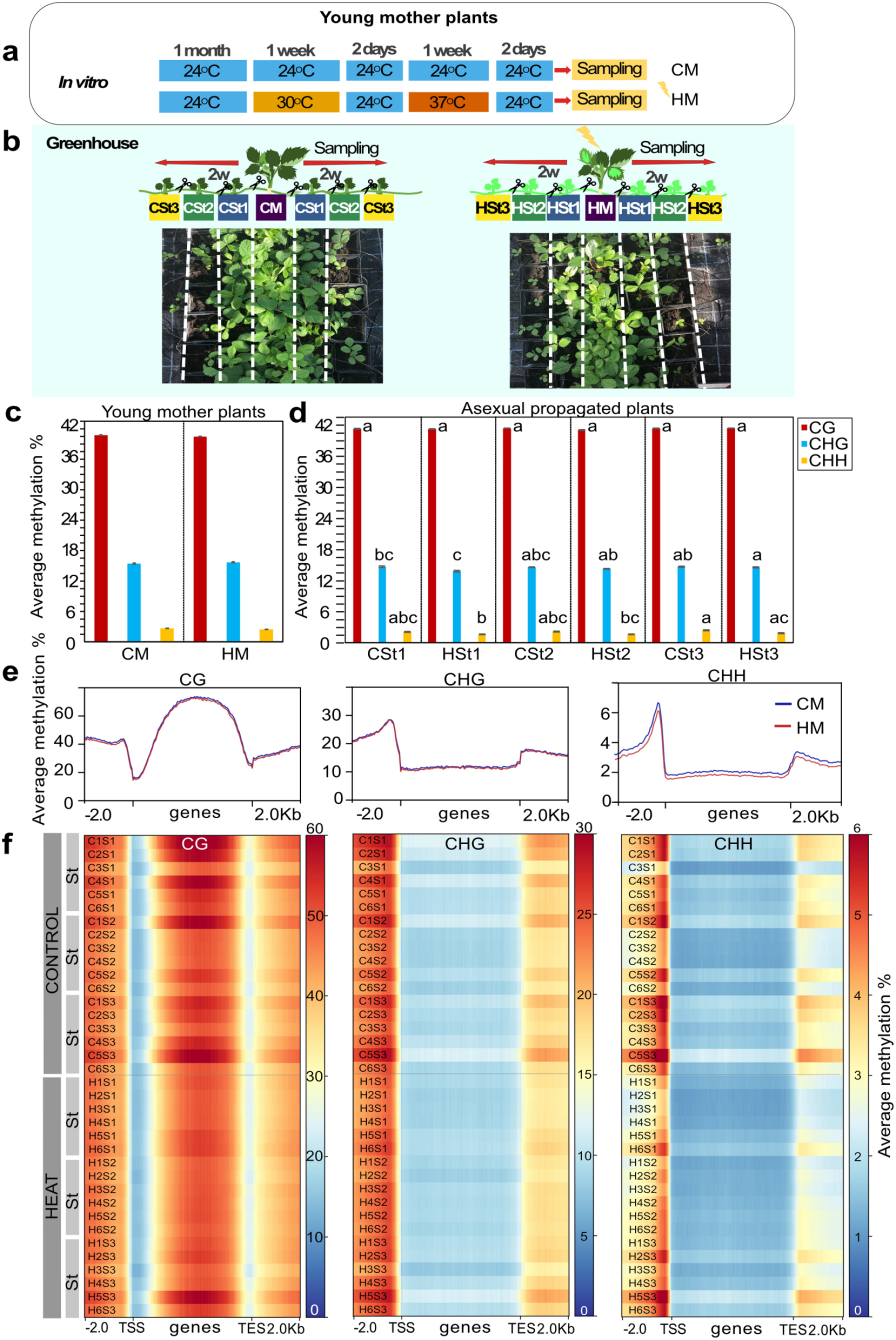
DNA methylation variability after heat stress and following asexual reproduction of *F. vesca*. **a** Scheme of the experimental design with treated and untreated plants. One-month-old strawberry seedlings treated with heat stress in vitro (pool of 5 plants; 3 replicates). CM: young control mother, HM: young heat-stressed mother. **b** Asexual reproduction *via* stolons (St) in the greenhouse. Photographs show two-week-old daughter plants produced by HM (*n* = 12) and CM (*n* = 12) plants. St1 (*n* = 24), St2 (*n* = 24), and St3 (*n* = 24): daughter plants of the first, second, and third asexual generations, respectively: CSt1 from CM, CSt2 from CSt1 and CSt3 from CSt2; HSt1 from HM, HSt2 from HSt1 and HSt3 from HSt2. **c** Average DNA methylation levels for each cytosine context (CG, CHG, CHH) between control (*n* = 6) and heat-stressed (*n* = 6) mother plants (not statistically significant, P value < 0.05); **d** asexual daughter plants per condition (*n* = 6 per group) (only common cytosine positions among all samples were considered that had a minimum coverage of 5 reads). Means not sharing a common letter in each asexual group are significantly different (Tukey’s test; P value < 0.05). **e** Plots showing the distribution of DNA methylation (top: CG, middle: CHG, and bottom: CHH) around genes of plants treated with (HM, in red) and without stress (CM, in blue). **f** Heatmaps showing the distribution of DNA methylation around genes of all daughter plants from each asexual generation and their replicates (*n* = 6). The average DNA methylation percentage (within a sliding 50-bp window) was plotted 2 kb upstream of TSS, over the gene body, and 2 kb downstream of TES

One-month-old seedlings were transferred to fresh MS media. For heat stress, plants were exposed to 30 °C (day/night) for one week, followed by two days of recovery (24 °C/21°C day/night) on fresh medium. The control plants went through the same recovery conditions. The plates were transferred to 37 °C (day/night) for one week followed by two recovery days (Fig. 1a). We sampled aerial parts of plants for molecular analyses. Three biological replicates of five pooled plants were collected per condition to reduce variability.

### Greenhouse propagation assays for daughter plants

After heat and control treatment, in vitro plants were transferred to soil (one plant per pot) in square plastic pots (size: 12 × 12 × 10 cm) and to a greenhouse with long day conditions (24 °C/21°C day/night and 60-70% humidity). Twelve mother plants (M) from control (CM; *n* = 12) and heat stress (HM; *n* = 12) conditions were propagated clonally for three generations (Fig. 1b). From each mother plant, the two first stolons (St1) were kept producing one daughter plant each in individual pots (Total St1 plants per condition = 24). After two weeks following root formation in St1, the stolons were cut to obtain independent plants from their mother plant (M). Next, from each daughter plant, CSt1 (*n* = 24) and HSt1 (*n* = 24), one stolon per plant was maintained to obtain the second asexual generation (St2). Similarly, the stolons were cut after two weeks to obtain independent daughter plants: CSt2 (*n* = 24) and HSt2 (*n* = 24). We kept the daughter plants to generate the third asexual generation (St3), and after two weeks, the stolon was cut to maintain independent daughter plants: CSt3 (*n* = 24) and HSt3 (*n* = 24) (Fig. 1b). All plants were kept until their reproductive phase (~ four-months-old) to collect seeds. For molecular analyses, the second new fully extended leaf from each independent daughter plant (12 plants per condition and per cycle) was sampled every two weeks for each asexual generation. For bisulfite and RNA sequencing, we pooled leaf samples collected from the two daughter plants from the same mother plant (*n* = 12). Six selected random samples were ground and separated in two tubes to obtain RNA (6 replicates per condition) and DNA (6 replicates per condition) from the same samples.

### DNA extraction and whole-genome bisulfite sequencing (WGBS) analysis

Genomic DNA was extracted with a plant DNA mini-kit, peqGOLD (VWR Life Science, cat#13-3486-01). Samples were sent to Novogene (Hongkong, China) for DNA library preparation and bisulfite sequencing. Paired-end reads were obtained on an Illumina (150 bp) NovaSeq6000 instrument. DNA methylation datasets were analyzed using the Epidiverse/wgbs pipeline [26] and the reference genome previously publish by López et al., 2022. Lambda DNA reads were used to evaluate the bisulfite conversion rate. An average of 61,257,191 reads (~ 30× coverage) were produced per sample. The bisulfite conversion rate was above 99.7% for all the samples (Additional file 1: Table S1). 81% of reads mapped properly to the *F. vesca* RV genome we previously sequenced [20]. Global DNA methylation levels were computed by combining all bedGraph files into a unionbedg file prefiltered for a minimum coverage of five reads per cytosine position and per sample. Only cytosine positions in common in all samples were kept. The R-package ggplot2 v.3.3.5 [27] was used for the visualization plots, and unpaired Student’s t-test was used for statistical analysis. P value < 0.05 was selected as the point of minimal statistical significance in all the analyses.

### Identification of differentially methylated regions (DMRs)

The previously pre-filtered bedGraph files obtained were used as input for the EpiDiverse/dmr bioinformatic analysis pipeline to identify DMRs [28] with default parameters. The parameters were: minimum coverage threshold 5; maximum q-value 0.05; minimum differential methylation level 10%; 10 as a minimum number of Cs; and minimum distance (bp) between Cs that are not to be considered as part of the same DMR is 146 bp. The pipeline uses metilene v.0.2.6.1 [29] (https://www.bioinf.uni-leipzig.de/Software/metilene/) for pairwise comparison between groups and R-packages ggplot2 v.3.3.5 [27] and gplots v.3.1.1 (https://CRAN.R-project.org/package=gplots), for visualization results (Additional file 2: Fig. S1). Based on our *F. vesca* genome transcript annotation and methylation data (overlapped regions with DNA methylation cytosines and DMRs), we detected the DMRs located in genic- and non-genic regions (genes, promoters, 3’ UTRs, 5’UTR, and TEs). Global DNA methylation and DMR plots were performed with R-package ggplot2 [27]. Gene analyses by methylation patterns and analysis of per-family TE DNA methylation profiles were performed with deepTools v.3.5.0 [30]. We produced genome browser tracks with DMRs in our publicly accessible JBrowse instance: https://jbrowse.agroscope.info/jbrowse/?data=fragaria_sub. To analyze common or shared DMR locations among all group of plants, we used the command bedtools [31] intersect in command-line: https://bedtools.readthedocs.io/en/latest/content/tools/intersect.html.

### RNA-seq analysis and definition of differentially expressed genes (DEGs)

Total RNA extractions were performed using a NucleoSpin RNA-Plus Mini-kit for RNA purification with a DNA removal column (cat# 740984.50). In vitro mother plant samples (CM; HM; *n* = 6) and daughter plant samples (St1; St2; St3; *n* = 36) were sent for Illumina paired-end read sequencing (150 bp) to Novogene (Hongkong, China). RNA-seq analyses were performed as described in [20, 32]. FastQC v.0.11.9 (https://www.bioinformatics.babraham.ac.uk/projects/fastqc/) and Trimmomatic v.0.39 (https://github.com/usadellab/Trimmomatic/releases) packages were briefly used for quality control and trimming. Salmon v1.4.0 [33] was used as a sequence mapper. The DESeq2 package [34] was used for quantitative differential gene transcription analysis on the European Galaxy platform (https://usegalaxy.eu) with default parameters (DEG: adjusted P value < 0.05) (Additional file 2: Fig. S2). Genes showing a DMR (promoter and gene-body located) and DEGs were annotated using Gene Ontology (GO) and Kyoto Encyclopedia of Genes and Genomes (KEGG) annotation downloaded from the Genome Database for Rosaceae (GDR) [35, 36]. Genes containing hypo- and hypermethylated DMRs were classified to investigate potential functions through GO enrichment by AgriGOv1.2 [37]. Similarly, DEGs were extracted with P adj. ≤0.05 for the GO functional analysis with Fisher’s exact test P values and Hochberg (FDR) as the multitest adjustment method (≤ 0.05 as the cut-off). For enriched pathways, KEGG analysis was performed with the R-package clusterProfiler [38]; pvalueCutoff = 0.05; pAdjustMethod="fdr”.

### Phenotyping assays

Five-month-old daughter plants of the three asexual generations from control and heat-stressed mother plants were treated with an ascending temperature gradient every 24 h (30 °C, 35 °C, 40 °C) to evaluate possible phenotypes and adaptation traits. Plants from the greenhouse were moved to plant growth chambers for heat stress treatment (*n* = 10 per condition). Leaf chlorophyll content was measured with a chlorophyll meter, SPAD-502Plus (KONICA MINOLTA), for three trifoliate opened leaves from each plant. Selected leaves were marked with a string to follow the variation in chlorophyll content with increasing temperature. A new group of younger daughter plants (one- month- old, *n* = 12 per condition) from a replicate experiment were exposed to 37 °C and 42 °C for 24 h to evaluate flowering time.

## Results

### Stress-induced DNA methylation differences across asexual strawberry plants

Following heat stress in vitro (Fig. 1a), we carried out a comprehensive analysis of DNA methylation in the three sequence contexts: CG, CHG, and CHH (H: A, T, or C). In heat-stressed young mother plants (HM, ~two- months-old), the global DNA methylation levels decreased by 1% in CG, 8% in CHH, and an increase by 2% in CHG contexts, compared to the young control mother plants (CM, ~two- months-old). However, these differences were not statistically significant (P <0.05, Fig. 1c). Methylome comparisons of young daughter plants from heat-stressed mothers (HSt1, HSt2, and HSt3; Fig. 1b) showed statistically significant (different letters, P <0.05) variability among groups for the non-CG sequence contexts compared to controls (CSt1, CSt2, and CSt3). The results are shown in Fig. 1d. To assess DNA methylation variation in genic and nongenic regions, the methylome data were screened in all three sequence contexts separately in three regions: 2 kb upstream of genes, over gene bodies, and 2 kb downstream of genes (total number: 34,007). We observed loss of CHH methylation in gene bodies and at the transcription start (TSS) and end (TES) sites when comparing treated and nontreated young mother plants (Fig. 1e). However, methylation in CG and CHG were stable across young mother plants (Fig. 1e). DNA methylation levels between CM-derived daughter plants showed variability whereas DNA methylation levels were stable in HM-derived daughter plants (Fig. 1f). DNA methylation variation over transposable element (TE) bodies and flanking regions exhibited distinct DNA methylation dynamics after heat stress particularly in the CHH context (Additional file 2: Fig. S3).

### Induction of persistent genomic DNA methylation variations by heat stress

**Fig. 2:**
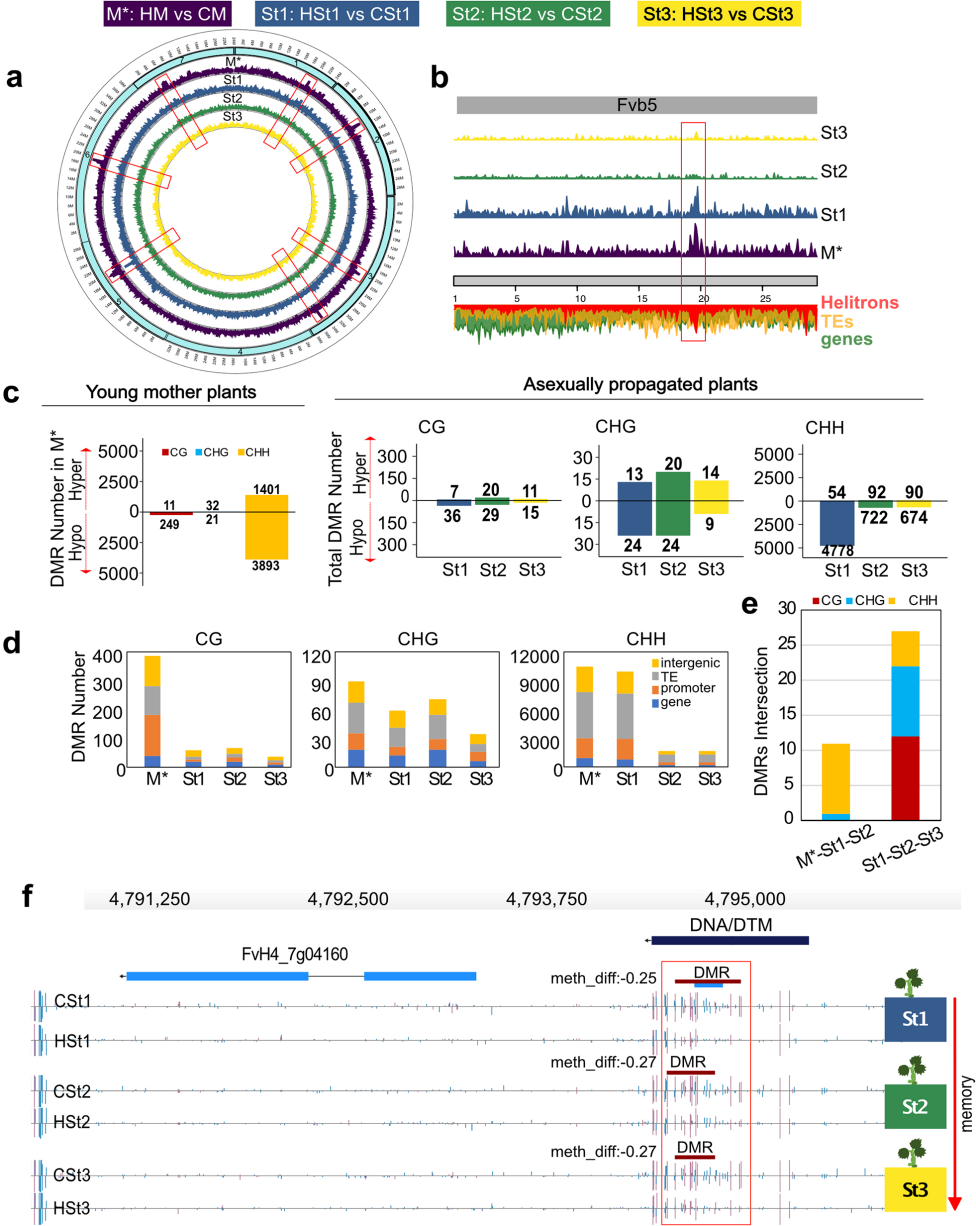
Transmission of heat stress-induced differentially methylated regions (DMRs) in *F. vesca* during asexual reproduction. **a** Circos plot showing the genome-wide distribution of DMR densities on all seven strawberry chromosomes (turquoise boxes) for each asexual plant generation. M*: young mother; St1, St2, and St3: daughter plants of the first, second, and third asexual generations, respectively. M*: HM (*n* = 3 pools) vs. CM (*n* = 3 pools), St1: HSt1 (*n* = 6) vs. CSt1 (*n* = 6), St2: HSt2 (*n* = 6) vs. CSt2 (*n* = 6), and St3: HSt3 (*n* = 6) vs. CSt3 (*n* = 6) comparisons (*: samples collected in vitro). **b** DMR density depicted for chromosome 5 (Fvb5) for all asexual generations. Below, the chromosome is indicated in gray, green shows gene density, yellow shows TE density and red shows *Helitron* density along this chromosome. **c** Total number of stress-induced hyper (hyperDMRs) and hypomethylated DMRs (hypoDMRs) are separated by sequence context. Differences between HM and CM in vitro are shown in the left plot. Daughter plant comparisons are shown in the right plot. **d** Distribution of DMRs in genomic features: promoter, gene-body, transposable elements (TE) and intergenic regions. Minimum overlap needed: 1 bp. **e** Bar plot depicting counts of common DMR locations (minimum overlap: 1 bp) containing hypo- and hyperDMRs per context among all populations. Boxes above the plot indicate the color codes for DNA methylation: red for CG, blue for CHG and yellow for CHH sequence contexts. **f** Genome browser views of overlapping DMRs located in the promoter region of a PR5-like receptor kinase gene (FvH4_7g04160) in St1, St2, and St3. Depicted are gene structures (top panels, UTRs in light blue, exons in blue), TEs (dark blue) and DNA methylation levels (histograms). Boxes above the histograms indicate identified DMRs with methylation difference ratios (color code for DNA methylation: red for CG)

We identified DNA methylation dynamics at specific loci, defining differentially methylated regions (DMRs) for each sequence context using the Epidiverse Toolkit with default parameters [28]. Samples were largely clustered according to each treatment group, separating control generations from heat-stressed generations (Additional file 2: Fig. S1). The distribution of differentially methylated region (DMR) densities across the seven chromosomes showed enrichment, particularly near centromeric and pericentromeric regions in the young mother plants comparisons (M*) (Fig. 2a). In addition, we identified the maintenance of DMR hotspots across the asexual generations, especially in St1, the first generation of plants not submitted to stress (Fig. 2b).

We counted the total number of DMRs for each group and sequence context (Fig. 2c). The majority of DMRs were detected in M* (HM vs. CM), with 5,607 DMRs. Reduced numbers of DMRs were found in the following asexual generations: 4,912 in St1, 907 in St2, and 813 in St3 (Fig. 2c). Moreover, we identified whether they were hypo- (hypoDMRs) or hypermethylated (hyperDMRs) for each sequence context. In the young mother plants, 74% of the DMRs were hypoDMRs. In the asexual generations, most DMRs were hypoDMRs such as 98% in St1; 85% in St2, and 86% in St3 relative to the control condition. To test whether genic and/or nongenic regions were enriched in DMRs, we quantified DMRs based on their intersection with promoters, gene bodies, and intergenic regions. In young mother plants, 37% of the CG-DMRs were in promoters, while in the asexual generations, between 29% and 36% of the CG-DMRs were in gene-body and intergenic regions (Fig. 2d). In all groups, between 35% and 50% of the CHG- and CHH-DMRs were over transposable elements (Fig. 2d). A gene ontology enrichment analysis with AgriGOv2 tool [37], from 2,745 genes with a DMR comparing HM vs. CM resulted in potential functional roles related to transport activity, localization process and cell membrane (Additional file 2: Fig. S4a). To further investigate the role of heat stress in inducing stable epigenetic changes in asexual generations, we analyzed whether DMRs present in asexual generations were acquired directly from heat-stressed parents. We found unique and common DMRs between St1, St2, St3, and M* (Fig. 2e). When considering all generations (M*-St1-St2-St3) together, there were no DMRs that were initially formed in M* and maintained consistently over three asexual generations. Interestingly, the first asexual generation (St1) retained the highest number of DMRs, 267 DMRs, among all the asexual generations. These DMRs were identified as CHH-DMRs (Additional file 2: Fig. S4b). In addition, 11 DMRs overlapped between M*, St1, and St2 (Fig. 2e). Comparisons of DMRs between generations showed 47% of the CG-DMRs, 37% of the CHG-DMRs and 9% of the CHH-DMRs in St1 that were maintained in at least one of the two subsequent asexual generations (Additional file 2: Fig. S4b). Notably, 27 DMRs were shared among all St generations (Fig. 2e, Additional file 1: Table S2). For example, we found a conserved DMR in the promoter region of PR5-like receptor kinase gene (FvH4_7g04160) in all HSt generations (Fig. 2f).

### Heat stress induced gene expression changes in stressed mother plants and non-stressed asexual generations

**Fig. 3:**
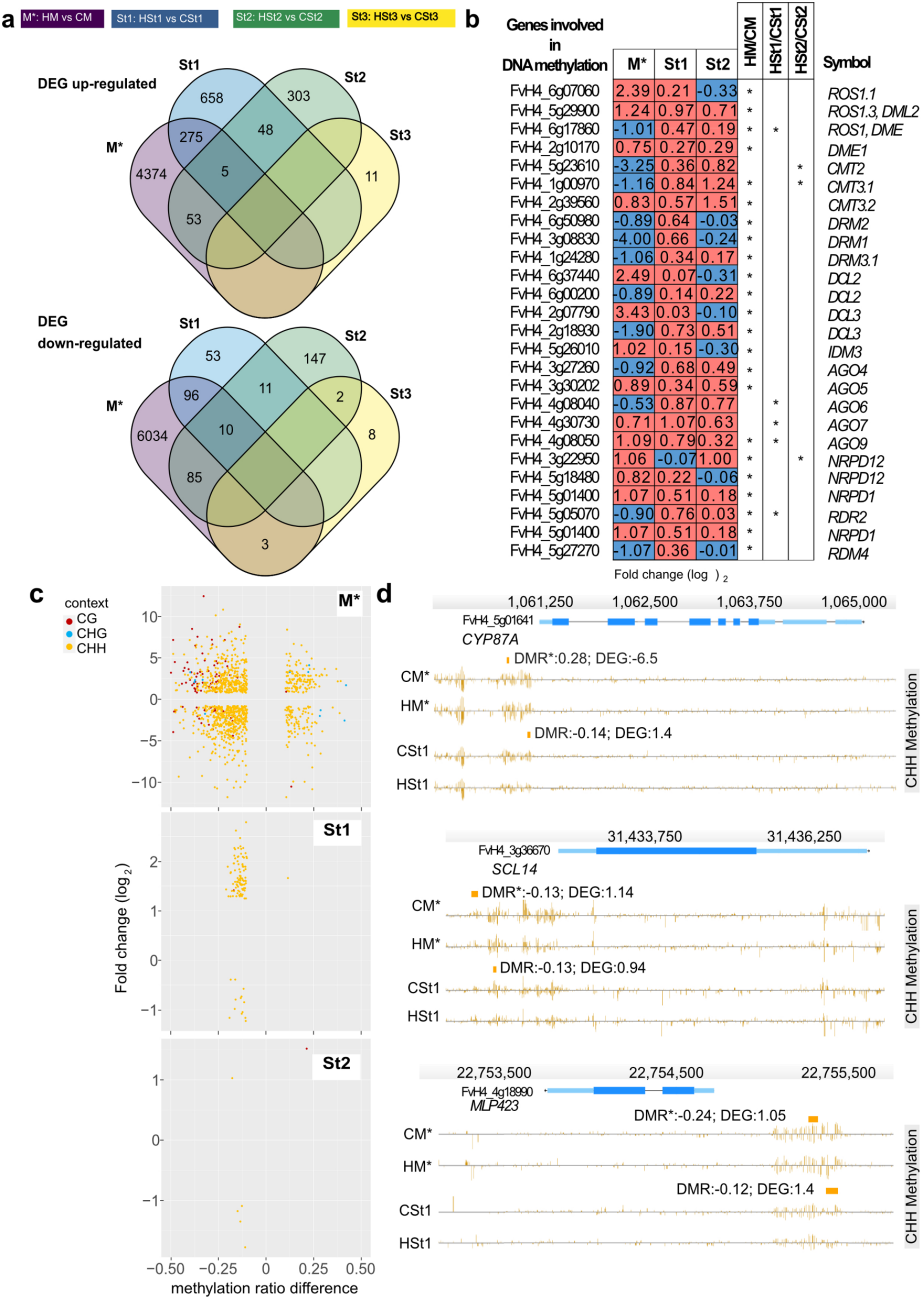
Heat stress induced differential gene expression in stressed mother plants and non-stressed asexual generations. **a** Venn diagrams displaying the intersections of differentially expressed genes of the four groups: after two days of recovery from young heat stress mother plants (M*) (*n* = 3 pools per condition) and two-week-old daughter plants (St1, St2, St3; *n* = 6 per conditions) (results from DESeq2, P adj. :<0.05). The top set represents upregulated genes, and the bottom set represents downregulated genes. **b** Transcriptional values (fold change in log_2_) of genes involved in DNA methylation and the RdDM pathway. * P adj. :<0.05. The first column shows the gene ID number, and the last column shows the gene symbols. **c** Scatterplots showing DEGs related to DMRs (promoter and gene body) in M* (upper plot); St1 (middle); and St2 (bottom). Points show the relationship between transcript levels (fold change on the y-axis) and DNA methylation (methylation difference on the x-axis). The color codes show methylation in CG red, in CHG blue and yellow for CHH contexts. **d** Genome browser views depicting selected DEGs after heat stress in M* and St1. The examples showed here are from top to bottom cytochrome P450 87A3-like (FvH4_5g01641), MLP-like protein 423 (FvH4_4g18990), and purple acid phosphatase 22 (FvH4_6g18411). Depicted are gene structures (top panels, UTRs in light blue, exons in blue), TEs (red and dark blue) and DNA methylation levels (histograms). Rectangles above the histograms indicate identified DMRs with DNA methylation difference ratios (color codes: red for CG, blue for CHG and yellow for CHH contexts). DEG designate transcription values in fold change (log_2_; results from DESeq2, P adj. :<0.05)

Transcriptome profiling, using the same material for methylation profiling, generated two clusters, one with M* plants and other with St1, St2, and St3 generations (Additional file 2: Fig. S4c). DEGs identified in M* plants exhibited higher fold change values in comparison to the St groups (Additional file 1: Table S3). Following heat stress, young mother plants displayed 10,935 DEGs (P adj. < 0.05), of which 57% were downregulated (Fig. 3a). Asexual generation comparisons (HSt vs. CSt) showed 1,156 in St1, 664 in St2 and 23 in St3 DEGs (P adj. < 0.05). In contrast to M* plants, the St1 and St2 generations mainly had upregulated DEGs: 85% in St1 and 62% in St2 (Fig. 3a, Additional file 1: Table S4, S5). Downstream analysis across the multiple asexual generations showed 28% of the up- and 56% of the downregulated genes (P adj. < 0.05) from St1 overlapped with those found in M*. Moreover, 13% of the up- and 33% of the downregulated genes (P adj. < 0.05) from St2 overlapped with in M* (Fig. 3a). In contrast, St3 presented the least transcriptional changes compared to the first two asexual generations, with no DEGs shared with M* (Additional file 1: Table S6). Despite the loss of most transcriptional changes over asexual generations, 15 DEGs (P adj. < 0.05) were maintained across two asexual reproductive cycles from M* to St1 and St2 (Fig. 3a). Gene ontology enrichment analyses (Additional file 2: Fig. S4d) in M* found biological processes such as metabolic process, response to stimulus, reproduction, macromolecule transport and modification. Additionally, there was enrichment in molecular functions such as transcription factor activity, regulation, and transferase activity (Additional file 2: Fig. S4d). The persistence of certain functions of the DEGs during different stages (St1 and St2) suggests a connection with processes like response to stress and stimulus in St1 (Additional file 2: Fig. S4e) and metabolic processes in St2 (Additional file 2: Fig. S4f). Most likely because of the reduced number of DEGs (P adj. < 0.05) in St3, no significant GO enrichment was detected. Pathway functional analysis (KEGG, P adj. < 0.05) for DEGs in M* plants showed enzymes related to reductase, monooxygenase, and transferase activity. St1 included transcription factors, peroxidase, and laccase activity. Laccase activity was also detected as a significant pathway in the St2 KEGG analysis (Additional file 2: Fig. S4g).

### Spontaneous and stable DNA methylation changes associated with gene expression patterns

Investigating direct effects of heat stress on the DNA methylation machinery, we identified genes coding for proteins involved in DNA demethylation (e.g., *ROS1* orthologs) were differentially expressed. Certain DNA methyltransferases (*CMT2, CMT3*) were downregulated in M*; however, they were upregulated in St1 and St2 (Fig. 3b). Members of the *ARGONAUTE* gene family, in volved in the RNA-directed DNA methylation pathway, were also differentially expressed. To illustrate, *AGO4* and *AGO6* were downregulated in M*, but *AGO6*, *AGO7* and *AGO9* were upregulated in St1. *NRPD12*, which encodes for the non-catalytic subunit common to nuclear DNA-dependent RNA polymerases II, IV and V was upregulated in M*. RNA-dependent RNA polymerase 2 (*RDR2*) and DICER-like genes were mostly upregulated only in M* (Fig. 3b).

Downstream analysis of the relationship between transcript and DNA methylation levels showed 492 DEGs (5% of the total DEGs) in M*, 94 DEGs in St1 (8%), and 16 DEGs in St2 (2%), that presented a DMR in their promoter or gene-body regions. We did not detect DEGs in St3 that were associated with DMRs. The presence of hypo- or hyperDMRs in the CHH context did not correlate with the observed gene expression patterns (Fig. 3c). Indeed, CHH-hypoDMRs were equally distributed between up- and downregulated genes in M*, St1 and St2 (Additional file 1: Table S7-S8). Remarkably in M*, 65 CG-hypoDMRs were related to upregulated genes (Additional file 1: Table S7) and 9 DEGs were correlated to five hypo- and four hyper- CHG-DMRs (Fig. 3c, Additional file 1: Table S7). We conducted Pearson correlation coefficient (PCC) analyses between DMRs and DEGs in M*, St1, and St2. In M*, a notably high negative correlation coefficient of -0.6659 was observed (Additional file 1: Table S7), indicating a moderate to strong negative correlation. This suggests that, in the M* stage, as DNA-methylation levels increase, transcription levels tend to decrease, and vice versa (Fig. 3c). In contrast, the correlation coefficients in St1 and St2 differed. St1 showed a weak positive correlation with a coefficient of 0.0425 (Additional file 1: Table S8), suggesting a negligible association between DMRs and DEGs. However, in St2, a relatively high positive correlation coefficient of 0.596 was observed (Additional file 1: Table S9), indicating a moderate to strong positive correlation. This suggests that, in St2, as DNA-methylation levels increase, transcription levels tend to increase, and vice versa. We identified 11 genes that maintained the DNA methylation difference and transcriptional profiles in M* and St1. For example, Fig. 3d shows candidate genes that might be epigenetically regulated and could be involved in stress memory. The cytochrome P450 family 87 gene (FvH4_5g01641, *CYP87*) had a CHH-DMR in the promoter region after heat stress in M*, with a DNA methylation ratio of 0.28 and a log_2_ of -6.5 for its differential expression. Similarly, in St1 plants, *CYP87* was associated with CHH-hypoDMRs and an increased transcript level (Fig. 3d). Because of differences in DNA methylation persisted over more than 60 days after heat stress (Fig. 2e), we wanted to assess the extent of the potential transmission of changes in DNA methylation and differential expression through asexual reproduction in daughter plants. We identified 20 genes related to one of the 27 stable DMRs in the St generations. These genes did not present significant expression differences (P adj. <0.05) in the St generations. However, 10 of the 20 genes were differentially transcribed in M* (P adj. <0.05). To illustrate, we show three examples (Additional file 2: Fig. S5) where heat stress treatment resulted in up- and downregulation of genes in M* that later acquired stable DMRs in the asexual generations.

### Testing transgenerational inheritance of heat stress-induced phenotypic traits in adult *F. Vesca* plants

**Fig. 4:**
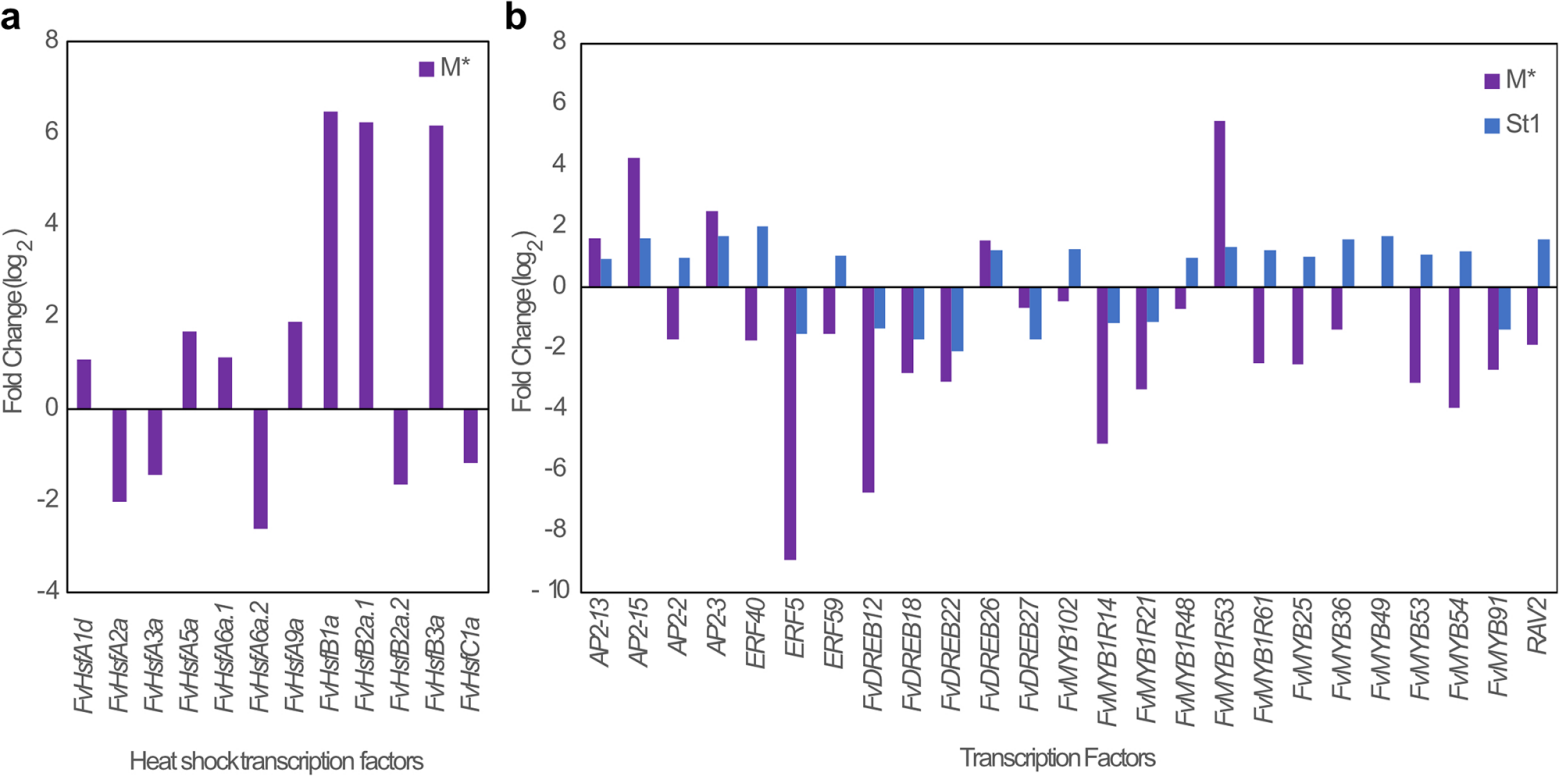
Effect of heat stress treatment on the expression of heat shock and transcription factors in stressed mother plants and non-stressed asexual generations. **a** Expression levels of Heat shock factors after heat stress in mother plants M* (HM vs. CM) values in fold change (log_2_; results from DESeq2, P adj. :<0.05). **b** APETALA2/ethylene-responsive response factors (*AP2/ERF*) and *MYB* family genes differentially expressed in stressed mother plants and non-stressed first asexual generation (log_2_; results from DESeq2, P adj. :<0.05)

We have previously demonstrated that heat stress resulted in genome-wide and local loss of DNA methylation and changed the expression of transcription factors (*TF*) [20]. In this study, from 19 annotated heat shock factors (*HSF)* genes, we found 12 *HSF* genes that were differentially expressed (P adj. < 0.05) in the mother plants after heat stress treatment (Fig. 4a, Additional file 1: Table S10); however, these *HFS* genes were not found to be differentially expressed in the non-stressed asexual generations. APETALA2/ethylene-responsive response factors (*AP2/ERF*) and *MYB* family genes were differentially expressed (P adj. < 0.05) in stressed mother plants and in the non-stressed first asexual generation (Fig. 4b Additional file 1: Table S11). Interestingly, we identified differential expression of 12 *TFs* showing a downregulation in M* and an upregulation in St1 (Fig. 4b). In addition, our analysis showed maintained expression profiles for 13 *TFs* (P adj. < 0.05) between the stressed mother plants and the non-stressed first asexual generation, indicating a prolonged transcriptional memory (Fig. 4b, Additional file 1: Table S11).

**Fig. 5:**
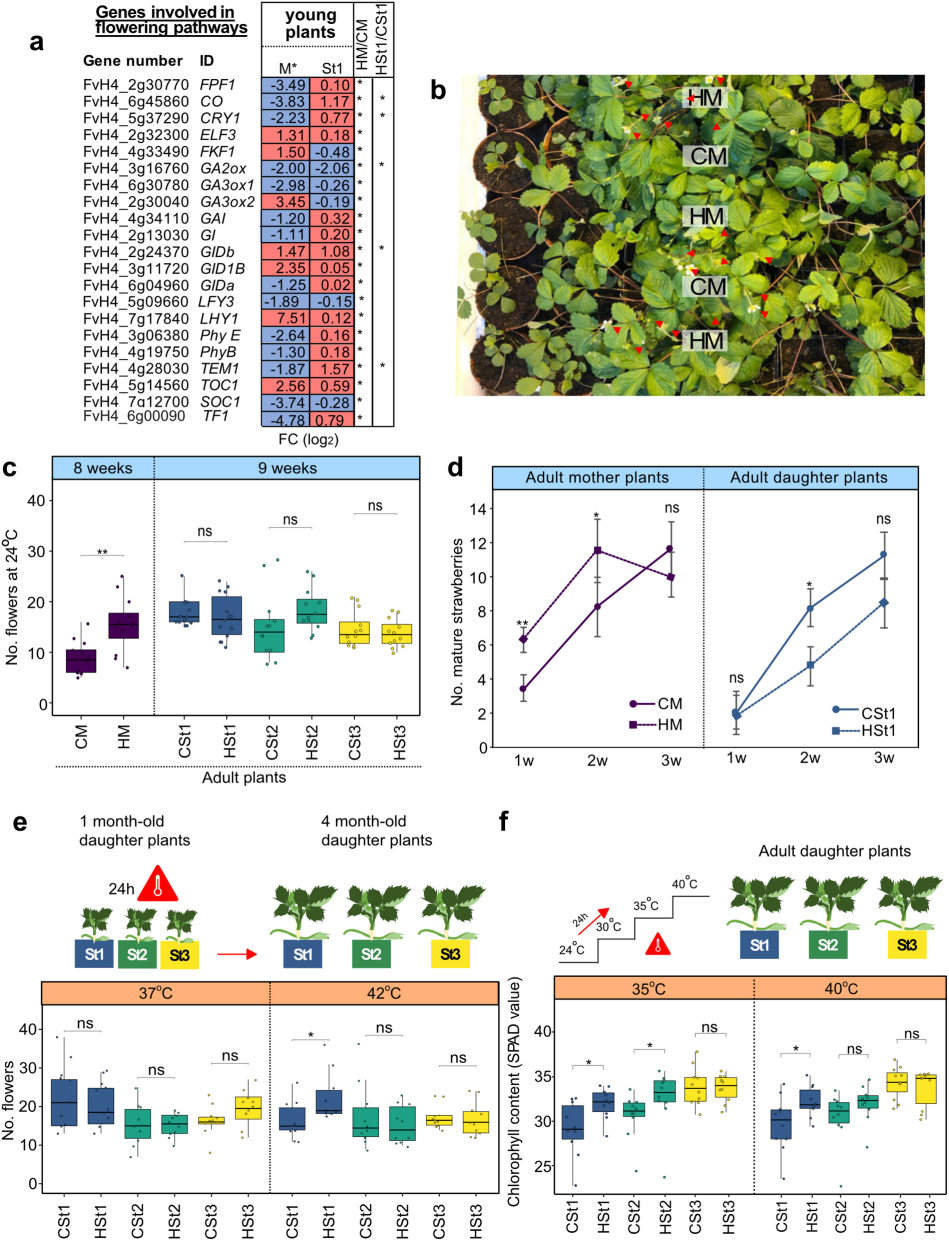
Phenotypes assessed over three clonal generations of plants issued from heat-stressed mother plants. **a** Table showing transcript levels (log_2_ change) of genes linked to flowering, including the photoperiod and gibberellin pathways, in M* (samples collected in vitro and St1, as determined using DESeq2). **b** Representative photograph of four-month-old mother plants from control (CM) and heat-stressed (HM) plants in a greenhouse during flowering. Red arrows point to closed and opened flowers. **c** Boxplots of total numbers of flowers per plant until the first ripe fruit was harvested in the greenhouse. **d** Number of harvested strawberries during three consecutive weeks in the adult M and St generations. **e** Total number of flowers per daughter plant exposed to a 24 h heat treatment (37 °C and 42 °C). **f** Total leaf chlorophyll content by the measurement of the Soil Pant Analysis Development (SPAD) values by chlorophyll meter of plants submitted to an ascending temperature gradient (+ 5 °C every 24 h). All statistical analyses were performed with Wilcoxon rank sum tests: *P value < 0.05; ns: not significant. HM: heat-stressed mother plant; CM: control mother plant; St1, St2, and St3: daughter plants of the first, second and third asexual generations, respectively

We detected changes in the expression of genes involved in flower regulation [39–41] during the young plant stages (Fig. 5a). To illustrate, the expression of *CONSTANS* (*CO*) and *TEMPRANILLO* (*TEM1)* between young mother plants, as well as in the young St1 generation had significant differences (P adj. < 0.05) compared to the control groups. Specifically, *CO* and *TEM1* were downregulated in M* and upregulated in St1 (Fig. 5d). Furthermore, our analysis revealed notable changes in genes associated with photoperiod and gibberellin pathways [42], including *CRYPTOCHROME 1* (*CRY1*), *GIBBERELLIN 2-OXIDASE* (*GA2ox*), and *GIBBERELLIN INSENSITIVE DWARF* (*GIDb*), in both M* and St1 generations. For instance, *CRY1* was downregulated in M*, whereas its transcript level significantly increased (P adj. < 0.05) in the St1 generation (Fig. 5a). *GA2ox* displayed a significant reduction in expression levels in both M* and St1 compared to control groups, while *GIDb* showed high expression levels in both M* and St1.

To assess the potential for heat stress to induce transmittable phenotypic changes, we monitored the development of HM and CM plants until adult stages under greenhouse conditions (Fig. 1b). HM adult plants showed an early flowering phenotype and produced an average of 16 flowers per plant compared to CM adult plants with an average of 9 flowers per plant (** P value < 0.01) (Fig. 5b, c). However, HSt1, HSt2, and HSt3 did not display significant differences (P value < 0.05) in flowering time when compared to the adult control generations.

We assessed fruit production by counting the number of mature strawberries. Initially, HM yielded a significantly (P value: ** < 0.01, * < 0.05) number of ripe strawberries, 6 and 10 fruits, during the first- and second-weeks post-flowering compared to 3 and 7 fruits in CM (Fig. 5d). However, by the third week, the number of harvested fruits decreased and became comparable to CM (Fig. 5d). Notably, during the second week, CSt1 presented an average of 6 fruit compared to HSt1 with an average of 4 fruit per plant (P value < 0.05, Fig. 5d). Across the subsequent two asexual generations (St2, St3), there were no significant differences (Additional file 2: Fig. S6a). Overall, the production of strawberries was similar in all the groups; nonetheless, there was earlier ripening of strawberries in HM which was directly related with the early flowering.

In addition, we noticed significant differences (P value < 0.05) in fruit size (width and height) with smaller fruits in the HM adult plants compared to the CM adult plants (Additional file 2: Fig. S6b). The dry biomass and number of seeds (Additional file 2: Fig. S6c) were not significantly different (P value < 0.05). To identify the heritability of heat stress effects on daughter plants at early stages, we performed a new heat stress experiment using one-month-old St populations to evaluate differences in flowering time at 37 °C and 42 °C for 24 h (Fig. 5e). We identified early flowering only after 42 °C in adult HSt1 plants (P value < 0.05), with an average of 23 flowers per plant, compared to adult CSt1 plants, with an average of 16 flowers per plant (Fig. 5e). There were no significant differences in the St2 and St3 groups.

To evaluate thermotolerance on adult daughter plants, we submitted St1, St2, and St3 adult plants to a rising gradient of temperature every 24 h from 24 °C to 40 °C. To assess leaf photochemical efficiency and leaf greenness, we measured chlorophyll content as SPAD (Soil Plant Analysis Development) values [43]. We detected significantly higher SPAD values in HSt1 and HSt2 at 35 °C and 40 °C compared to their corresponding controls (Fig. 5f; Additional file 2: Fig. S6d). For example, CSt1 leaves had 29 SPAD values at 35 °C and 40 °C. However, HSt1 leaves had 32 SPAD on average at 35 °C and 40 °C (P value < 0.05, Fig. 5f). To summarize, only the immediate clonal daughter plants (HSt1) from heat-treated plants (HM) showed significant phenotypic variability compared control plants.

## Discussion

Genetic mutations can lead to the emergence of favorable and unfavorable traits which will be selected for or against by natural selection or breeding. However, both genetic mutations and epimutations can contribute to plant diversity [44–46]. Plant epigenomes have been examined after unfavorable environmental conditions to evaluate how epigenetic changes trigger adaptive responses [47]. High temperature is one of the most studied environmental stress conditions due to its negative effects on natural plant populations and crops. To identify physiological changes which can affect plant yield and quality, genetic and epigenetic studies in Arabidopsis have been performed under heat stress. These studies showed how dynamic DNA methylation, histones modification and small interfering RNAs (siRNA) are under heat stress and how these changes are correlated with changes in transcription and activation of transposable elements (TEs) [48–53]. Even though epigenetic mechanisms are conserved among plant species [54], plants can differ in the way they perceive and respond to high temperatures. For instance, soybean roots and rapeseed seedlings under heat treatment showed hypomethylation in all cytosine contexts [55, 56]. The development and maintenance of a memory which can be maintained through mitosis and meiosis will depend on different factors: plant genotype, type of the external stimuli, intensity, and recurrence of stress [57]. We have previously shown that heat stress resulted in the most significant reduction of DNA methylation in *F. vesca* [20] when compared to other types of stresses while in the case of asexually reproducing dandelions hormonal stress caused mayor changes in DNA methylation compared to other abiotic stress conditions [58]. Similarly, drought stress has been documented to affect the epigenome of plants such as poplar, mulberry and sesame [59–62].

To investigate the heritability of stress-induced epigenetic changes in *F. vesca*, we analyzed the methylomes of young daughter plants across three successive asexual generations (St1, St2 and St3). These plants were raised in a common control environment, with the variation being the young mother plants that were either submitted to heat stress conditions or not. We observed predominantly hypoDMRs across all cytosine contexts, which were particularly notable in TSS and TES genomic regions (Fig. 2c). Similar results were found in tobacco, maize, and seagrasses where reduction of DNA methylation was mostly identified over promoter regions and gene bodies during heat stress [63–65]. Overall, we identified 10,935 differentially expressed genes (DEGs) and 5,607 differentially methylated regions (DMRs) in mother plants after the stress treatment. From these group of genes, 492 DEGs were directly associated with a DMR in their promoter or gene-body regions. These results suggest there is an interconnected mechanism which regulates transcription and DNA methylation in a specific manner during stress (Fig. 3c). For example, genes related with a CG DMR predominantly showed a negative correlation with their expression patters. In general, if a gene was associated with a CG hypomethylated DMR, the expression was upregulated (Fig. 3c). It has been shown in Arabidopsis, that the role of CG methylation on gene expression is much stronger than non-CG methylation because it recruits sequence specific transcription factors which are essential for tissue specific gene expression [66, 67].

A limited number of heat stress-induced DMRs and DEGs from the maternal generation (M*) were detected in the first generation (St1), including 267 DMRs (Additional file 2: Fig. S4b) and 386 DEGs (Fig. 3a). The St1 asexual generation consists of daughter plants originating from the first two stolons of the mother plant, which are generated from axillary meristems (AXM) [68]. In the near-isogenic *F. vesca* line used in this study, the first two stolons emerged from the AXM of the sixth adult trifoliate leaf [69]. The leading hypothesis suggest that meristematic cells, which later develop into axillary meristems, experience heat stress, and retained a molecular memory of this stress. However, with each subsequent asexual generation, characterized by numerous cell divisions, this memory gradually diminishes. Studies of meristematic cell divisions have shown how genetic and epigenetic variations occur in cells and how they contribute to new tissues [9, 70]. Mutations are suggested to occur in cells of the apical meristem and be maintained in the new shoot-meristem, whereas changes in subapical meristematic cells are easily displaced [9]. Similarly, epigenetic variations in meristematic cells can lead to epigenetic mosaicisms during the growth and branch development of wild lavender (*Lavandula latifolia*), which might persist in an ecological and evolutionary manner [71].

Upon examining all *F. vesca* generations collectively (M*-St1-St2-St3), we found no DMRs and DEGs that were directly induced in M* by heat stress and retained directly over three consecutive asexual generations (Figs. 2e and 3a, Additional file 2: Fig. S4b). The reduced differences in DNA methylation and transcription in St3 showed that without the presence of the heat stimuli, plants generally recover the initial DNA methylation patterns. It has been shown that a constant external stimulus is required to induce longer lasting memory. For instance, salt, lack of water, iron deficiency, spaceflight and UV-C stress conditions have been reported to induce long-term memory that could last over several generations (from F2 to F5) after continuous stress exposure [24, 72–75]. In duckweed (*Lemma minor* L.), twelve clonal generations exposed to 30 °C for nine weeks showed long-term epigenetic memory in CG and CHG loci containing hypermethylated regions [76]. Even though wild-type DNA methylation patterns were largely restored in our experiments, we identified 27 fixed DMRs persisting across all asexual generations and biological replicates (*n* = 6 per condition). This robust presence strongly indicates a degree of establishment and transgenerational inheritance of heat stress-induced DNA methylation changes, effectively documenting the occurrence of stress-induced epimutations (Fig. 2e). Therefore, these loci represent heat stress responsive epimutable loci. For instance, DNA methylation analysis in *NRPD1* mutant Arabidopsis plants identified hypomethylated regions which were categorized in regions which were able to restored methylation in the presence of NRPD1 and those that kept the hypomethylation state for more than 20 generations [77]. This specific behavior has been associated with high levels of histone marks, including H3K4m3 and H3K18ac, which prevent the recruitment of the RdDM machinery [77]. Understanding the molecular signatures that delineate these epimutable loci holds significant interest and warrants further investigation.

Building on the understanding that heat stress triggers rapid responses (from seconds to minutes) and rapid systemic signaling pathways to regulate gene expression [78, 79], our study investigated the potential mechanisms underlying stress-induced DNA methylation changes. Specifically, we identified differentially expressed genes involved in several DNA methylation pathways across multiple generations (M*, St1 and St2) (Fig. 3a), suggesting a potential contribution of pathway perturbations to stochastic DNA methylation alterations. There is evidence demonstrating how high temperatures can generate instability in plant genomes and cause over expression of heat shock factors (*HSF*) and heat shock proteins (*Hsp*) [80–82]. In the case of tomato, plants showed specific conformations of chromatin states after heat stress and displayed interactions between the heat shock transcription factor HSFA1a and promoter enhancers controlling the expression of stress-response genes [83]. In strawberry after heat stress, we found *HSFA1d* and *HSFA5a* to be upregulated in M* (Fig. 4a). Therefore, heat stress likely triggered transcriptional changes followed by modifications in DNA methylation marks. Supporting this hypothesis, we identified changes in gene expression between HM and CM that later overlapped with the formation of stable DMRs in the asexual generations (Additional file 2: Fig. S5). Nonetheless, most DEGs were not linked to DNA methylation changes. Some DEGs were associated to changes in DNA methylation in their promoters and TESs in M* and this status was maintained until St1 (Fig. 3d). Similarly, phosphate starvation appeared to first activate stress response genes, and as a consequence, DNA methylation differences were induced [22]. In addition, the reduction in DNA methylation by the chromatin remodeler *DDM1* in poplar lines showed that plants were more tolerant to drought conditions because of the activation of stress-responsive genes [84].

In addition, we observed downregulation of genes involve in the flowering pathway. To illustrate, *CO* and *TEM1* in the young mother plants (M*) followed by upregulation in the St1 generation suggesting a dynamic regulation of these genes across generations, possibly influenced by epigenetic factors (Fig. 5a). Furthermore, the significant reduction in expression levels of *GA2ox* in both HM* and HSt1 groups, coupled with notably high expression levels of *GIDb* in these groups, indicates consistent expression profiles of these gibberellin-linked genes across M* and St1 generations. This suggests a potential contribution of these genes to short transcriptional memory and regulation of flowering time control. These findings align well with previous research on *TEM* genes in *Fragaria vesca* and apple (*Malus domestica*) [39, 41]. To illustrate, *in Fragaria vesca* plants, the RNAi silencing of *TEM1 (RNAi-TEM)* resulted in five days earlier flowering compared to lines where strawberries were transformed to overexpress apple *MdTEM1* and *MdTEM2*, leaded to downregulation of gibberellin genes and delayed flowering [41]. In addition, heat stress has previously been described as a factor that stimulates early flowering, and that this phenotypic trait can be inherited over several generations in Arabidopsis [85, 86]. Stress-induced flowering is considered a survival sign since seed production will guarantee the species prevalence [87]. We confirmed that *F. vesca* submitted to heat stress had shorter flowering times compared to control plants (Fig. 5c). A comparable phenotype was observed in the stressed HSt1 generation at 42 °C regardless of whether the CSt1 generation was submitted to the heat treatment or not (Fig. 5e). Thermotolerance can be evaluated measuring membrane damage, maximum photochemical efficiency of photosystem II (*Fv/Fm*), chlorophyll content, seed viability or the production of reactive oxygen species (ROS) in plant tissues [88]. Loss of chlorophyll and performance of the photosystem II in *Suaeda salsa*, maize and rice was a phenotype associated with heat tolerance [89, 90]. Reduced chlorophyll content due to heat stress is known to affect productivity of strawberry cultivars [91, 92]. We found that under heat stress, HSt1 maintained the chlorophyll content as compared to CSt1 (Fig. 5f), suggesting that HSt1 may cope better with a subsequent heat stress.

Our study offers valuable insights into the dynamics of epigenetic and transcriptional changes, shedding light on the mechanisms underlying plant memory maintenance and transmission across generations. Understanding these processes is crucial for unraveling the intricacies of stress memory formation and transmission in plants, holding significant implications for boosting resilience and productivity in agricultural contexts.

Moving forward, a critical next step involves investigating whether heritable stress-induced epigenetic alterations contribute to stress adaptation. This can be achieved through targeted analyses of DNA methylation modifications and by conducting plant competition experiments in natural environments. Such investigations will provide deeper insights into the adaptive potential of plants and inform strategies for optimizing stress response mechanisms in agricultural practices.

## Conclusions

Our findings contribute to elucidating the molecular mechanisms underlying stress memory acquisition and maintenance in *Fragaria vesca*. We have demonstrated that early exposure to stress can induce significant alterations in the epigenome and transcriptome of plants, potentially facilitating some level of transfer of information across clones. Notably, our study identified instances of heat stress-induced epimutation formation, suggesting adaptive responses that may influence asexual generations probably enhancing their ability to cope with subsequent stressors. Furthermore, our research highlights the cumulative nature of epigenetic and transcriptomic changes triggered by heat stress, documenting the formation of a molecular memory. This memory could play a role in shaping the responses of future generations to stress conditions. These findings underscore the dynamic nature of the maternal epigenome and transcriptome, which can effectively modulate signaling pathways and behavior in asexual progenies under future stress environments.

### Electronic supplementary material

Below is the link to the electronic supplementary material.

Supplementary Material 1

Supplementary Material 2

## Data Availability

The datasets generated and/or analyzed in this study are available in the Zenodo repository DOI: 10.5281/zenodo.7898322. All the sequencing data from this study are at the European Nucleotide Archive (ENA, www.ebi.ac.uk/ena/, ERP136614). The data can be accessed under the project PRJEB51950. Bisulfite-sequencing raw read fastq accessions: ERS11389778- ERS11389819. Raw reads of the RNA-sequencing under the accessions: ERS15458182- ERS15458217.

## Abbreviations

CM: control mother plant
CSt1: control stolon 1
CSt2: control stolon 2
CSt3: control stolon 3
HM: heat-stressed mother plant
HSt1: Stolon 1 from a heat-stressed mother
HSt2: Stolon 2 from a heat-stressed mother
HSt3: Stolon 2 from a heat-stressed mother
WGBS: whole-genome bisulfite sequencing
CHGH: Adenine (A), Thymine (T), or Cytosine (C)
gbM: body methylation genes
M*: comparison of young mother plants grown in vitro (HM vs. CM)
St1: comparison HSt1 vs. CSt1
St2: comparison HSt2 vs. CSt2
St3: comparison HSt3 vs. CSt3

## References

[1] Zhang H, Zhu J, Gong Z, Zhu JK. Abiotic stress responses in plants. Nat Rev Genet. 2021;0123456789. 10.1038/s41576-021-00413-0 34561623

[2] He Y, Chen T, Zeng X. Genetic and epigenetic understanding of the Seasonal timing of Flowering. Plant Commun. 2020;1(1). 10.1016/j.xplc.2019.100008 PMC7747966 33404547

[3] Battey NH (2000). Aspects of seasonality. J Exp Bot.

[4] Wang R, Farrona S, Vincent C, Joecker A, Schoof H, Turck F (2009). PEP1 regulates perennial flowering in *Arabis alpina*. Nature.

[5] Yang YY, Kim JG. The optimal balance between sexual and asexual reproduction in variable environments: a systematic review. J Ecol Environ. 2016;40(1).

[6] Price EAC, Marshall C. Clonal plants and environmental heterogeneity: An introduction to the proceedings. Plant Ecol. 1999;141(1–2):3–7.

[7] García-Verdugo C, Calleja JA, Vargas P, Silva L, Moreira O, Pulido F (2013). Polyploidy and microsatellite variation in the relict tree Prunus lusitanica L.: how effective are refugia in preserving genotypic diversity of clonal taxa?. Mol Ecol.

[8] McKey D, Elias M, Pujol ME, Duputié A (2010). The evolutionary ecology of clonally propagated domesticated plants. New Phytol.

[9] Burian A, Barbier de Reuille P, Kuhlemeier C (2016). Patterns of Stem Cell divisions Contribute to Plant Longevity. Curr Biol.

[10] Jiang C, Mithani A, Belfield EJ, Mott R, Hurst LD, Harberd NP (2014). Environmentally responsive genome-wide accumulation of de novo *Arabidopsis thaliana* mutations and epimutations. Genome Res.

[11] Wibowo A, Becker C, Durr J, Price J, Spaepen S, Hilton S (2018). Partial maintenance of organ-specific epigenetic marks during plant asexual reproduction leads to heritable phenotypic variation. Proc Natl Acad Sci U S A.

[12] Wang L, Ji Y, Hu Y, Hu H, Jia X, Jiang M (2019). The architecture of intra-organism mutation rate variation in plants. PLoS Biol.

[13] Cao Q, Feng Y, Dai X, Huang L, Li J, Tao P (2021). Dynamic changes of DNA methylation during Wild Strawberry (*Fragaria Nilgerrensis*) tissue culture. Front Plant Sci.

[14] Bairu MW, Aremu AO, van Staden J (2011). Somaclonal variation in plants: causes and detection methods. Plant Growth Regul.

[15] Sammarco I, Münzbergová Z, Latzel V. DNA methylation can mediate local adaptation and response to Climate Change in the Clonal Plant Fragaria vesca : evidence from a european-scale. Recipr Transpl Exp. 2022;13(February). 10.3389/fpls.2022.827166 PMC8919072 35295625

[16] De Kort H, Panis B, Deforce D, Van Nieuwerburgh F, Honnay O (2020). Ecological divergence of wild strawberry DNA methylation patterns at distinct spatial scales. Mol Ecol.

[17] Galanti D, Ramos-Cruz D, Nunn A, Rodríguez-Arévalo I, Scheepens JF, Becker C, Bossdorf O. Genetic and environmental drivers of large-scale epigenetic variation in *Thlaspi arvense*. PLoS genetics. 2022 Oct 12;18(10):e1010452. 10.1371/journal.pgen.1010452 PMC9591053 36223399

[18] Dubin MJ, Zhang P, Meng D, Remigereau MS, Osborne EJ, Casale FP (2015). DNA methylation in Arabidopsis has a genetic basis and shows evidence of local adaptation. Elife.

[19] Zhang Y, Fan G, Toivainen T, Tengs T, Yakovlev I, Krokene P et al. Warmer temperature during asexual reproduction induce methylome, transcriptomic and lasting phenotypic changes in *Fragaria vesca* ecotypes. Hortic Res. 2023;uhad156. 10.1093/hr/uhad156 PMC10500154 37719273

[20] López M-E, Roquis D, Becker C, Denoyes B, Bucher E. DNA methylation dynamics during stress response in woodland strawberry (*Fragaria vesca*). Hortic Res. 2022;15261. 10.1093/hr/uhac174 PMC9533225 36204205

[21] Kou S, Gu Q, Duan L, Liu G, Yuan P, Li H et al. Genome-wide bisulphite sequencing uncovered the contribution of DNA methylation to Rice Short-Term Drought memory formation. J Plant Growth Regul. 2021;(0123456789).

[22] Secco D, Wang C, Shou H, Schultz MD, Chiarenza S, Nussaume L (2015). Stress induced gene expression drives transient DNA methylation changes at adjacent repetitive elements. Elife.

[23] Rendina González AP, Preite V, Verhoeven KJF, Latzel V (2018). Transgenerational effects and epigenetic memory in the clonal plant trifolium repens. Front Plant Sci.

[24] Wibowo A, Becker C, Marconi G, Durr J, Price J, Hagmann J, et al. Hyperosmotic stress memory in Arabidopsis is mediated by distinct epigenetically labile sites in the genome and is restricted in the male germline by DNA glycosylase activity eLife. 2016;5:e13546. 10.7554/eLife.13546 PMC4887212 27242129

[25] Urrutia M, Bonet J, Arús P, Monfort A (2015). A near-isogenic line (NIL) collection in diploid strawberry and its use in the genetic analysis of morphologic, phenotypic and nutritional characters. Theor Appl Genet.

[26] Nunn A, Otto C, Stadler PF, Langenberger D (2021). Comprehensive benchmarking of software for mapping whole genome bisulfite data: from read alignment to DNA methylation analysis. Brief Bioinform.

[27] Wickham H. ggplot2: Elegant Graphics for Data Analysis. Springer-Verlag New York. Vol. 35, Media. 2016. 211 p.

[28] Nunn A, Can SN, Otto C, Fasold M, Stadler PF, Langenberger D. EpiDiverse Toolkit : a pipeline suite for the analysis of bisulfite sequencing data in ecological plant epigenetics. 2021;3(4):1–7. 10.1093/nargab/lqab106 PMC8598301 34805989

[29] Jühling F, Kretzmer H, Bernhart SH, Otto C, Stadler PF, Hoffmann S (2016). Metilene: fast and sensitive calling of differentially methylated regions from bisulfite sequencing data. Genome Res.

[30] Ramírez F, Dündar F, Diehl S, Grüning BA, Manke T (2014). DeepTools: a flexible platform for exploring deep-sequencing data. Nucleic Acids Res.

[31] Quinlan AR, Hall IM, BEDTools (2010). A flexible suite of utilities for comparing genomic features. Bioinformatics.

[32] Roquis D, Robertson M, Yu L, Thieme M, Julkowska M, Bucher E (2021). Genomic impact of stress-induced transposable element mobility in Arabidopsis. Nucleic Acids Res.

[33] Patro R, Duggal G, Love MI, Irizarry RA, Kingsford C (2017). Salmon provides fast and bias-aware quantification of transcript expression. Nat Methods.

[34] Love MI, Huber W, Anders S (2014). Moderated estimation of Fold change and dispersion for RNA-seq data with DESeq2. Genome Biol.

[35] Jung S, Lee T, Cheng CH, Buble K, Zheng P, Yu J (2019). 15 years of GDR: New data and functionality in the genome database for Rosaceae. Nucleic Acids Res.

[36] Li Y, Pi M, Gao Q, Liu Z, Kang C. Updated annotation of the wild strawberry *Fragaria vesca* V4 genome. Hortic Res. 2019;6(1). 10.1038/s41438-019-0142-6 PMC6491553 31069085

[37] Tian T, Liu Y, Yan H, You Q, Yi X, Du Z (2017). AgriGO v2.0: a GO analysis toolkit for the agricultural community, 2017 update. Nucleic Acids Res.

[38] Wu T, Hu E, Xu S, Chen M, Guo P, Dai Z (2021). clusterProfiler 4.0: a universal enrichment tool for interpreting omics data. Innov.

[39] Gaston A, Potier A, Alonso M, Sabbadini S, Delmas F, Tenreira T (2021). The FveFT2 florigen/FveTFL1 antiflorigen balance is critical for the control of seasonal flowering in strawberry while FveFT3 modulates axillary meristem fate and yield. New Phytol.

[40] Kurokura T, Samad S, Koskela E, Mouhu K, Hytönen T (2017). Fragaria vesca CONSTANS controls photoperiodic flowering and vegetative development. J Exp Bot.

[41] Dejahang A, Maghsoudi N, Mousavi A, Farsad-Akhtar N, Matias-Hernandez L, Pelaz S (2023). TEMPRANILLO homologs in apple regulate flowering time in the woodland strawberry Fragaria vesca. Sci Rep.

[42] Guo X, Xie Z, Zhang Y, Wang S (2021). The FvCYP714C2 gene plays an important role in gibberellin synthesis in the woodland strawberry. Genes Genomics.

[43] Xiong D, Chen J, Yu T, Gao W, Ling X, Li Y (2015). SPAD-based leaf nitrogen estimation is impacted by environmental factors and crop leaf characteristics. Sci Rep.

[44] Quadrana L, Colot V (2016). Plant Transgenerational epigenetics. Annu Rev Genet.

[45] Zamir D (2001). Improving plant breeding with exotic genetic libraries. Nat Rev Genet.

[46] Schmitz RJ, Schultz MD, Lewsey MG, O’Malley RC, Urich MA, Libiger O (2011). Transgenerational epigenetic instability is a source of novel methylation variants. Sci (80-).

[47] Verhoeven KJF, VonHoldt BM, Sork VL (2016). Epigenetics in ecology and evolution: what we know and what we need to know. Mol Ecol.

[48] Naydenov M, Baev V, Apostolova E, Gospodinova N, Sablok G, Gozmanova M (2015). High-temperature effect on genes engaged in DNA methylation and affected by DNA methylation in Arabidopsis. Plant Physiol Biochem.

[49] Ito H, Gaubert H, Bucher E, Mirouze M, Vaillant I, Paszkowski J (2011). An siRNA pathway prevents transgenerational retrotransposition in plants subjected to stress. Nature.

[50] Popova OV, Dinh HQ, Aufsatz W, Jonak C (2013). The RdDM pathway is required for basal heat tolerance in arabidopsis. Mol Plant.

[51] Song ZT, Zhang LL, Han JJ, Zhou M, Liu JX, Histone (2021). H3K4 methyltransferases SDG25 and ATX1 maintain heat-stress gene expression during recovery in Arabidopsis. Plant J.

[52] Bäurle I, Trindade I (2020). Chromatin regulation of somatic abiotic stress memory. J Exp Bot.

[53] Liu J, Feng L, Gu X, Deng X, Qiu Q, Li Q (2019). An H3K27me3 demethylase-HSFA2 regulatory loop orchestrates transgenerational thermomemory in Arabidopsis. Cell Res.

[54] Niederhuth CE, Bewick AJ, Ji L, Alabady MS, Kim K, Do, Li Q (2016). Widespread natural variation of DNA methylation within angiosperms. Genome Biol.

[55] Hossain MS, Kawakatsu T, Kim K, Do, Zhang N, Nguyen CT, Khan SM (2017). Divergent cytosine DNA methylation patterns in single-cell, soybean root hairs. New Phytol.

[56] Gao G, Li J, Li H, Li F, Xu K, Yan G (2014). Comparison of the heat stress induced variations in DNA methylation between heat-tolerant and heat-sensitive rapeseed seedlings. Breed Sci.

[57] Lloyd JPB, Lister R. Epigenome plasticity in plants. Nat Rev Genet. 2021;0123456789. 10.1038/s41576-021-00407-y 34526697

[58] Verhoeven KJF, Jansen JJ, van Dijk PJ, Biere A (2010). Stress-induced DNA methylation changes and their heritability in asexual dandelions. New Phytol.

[59] Komivi D, Marie AM, Rong Z, Qi Z, Mei Y, Ndiaga C (2018). The contrasting response to drought and waterlogging is underpinned by divergent DNA methylation programs associated with transcript accumulation in sesame. Plant Sci.

[60] Rico L, Ogaya R, Barbeta A, Peñuelas J (2014). Changes in DNA methylation fingerprint of Quercus ilex trees in response to experimental field drought simulating projected climate change. Plant Biol.

[61] Li R, Hu F, Li B, Zhang Y, Chen M, Fan T (2020). Whole genome bisulfite sequencing methylome analysis of mulberry (Morus alba) reveals epigenome modifications in response to drought stress. Sci Rep.

[62] Lafon-Placette C, Le Gac AL, Chauveau D, Segura V, Delaunay A, Lesage-Descauses MC (2018). Changes in the epigenome and transcriptome of the poplar shoot apical meristem in response to water availability affect preferentially hormone pathways. J Exp Bot.

[63] Entrambasaguas L, Ruocco M, Verhoeven KJF, Procaccini G, Marín-Guirao L (2021). Gene body DNA methylation in seagrasses: inter- and intraspecific differences and interaction with transcriptome plasticity under heat stress. Sci Rep.

[64] Centomani I, Sgobba A, D’Addabbo P, Dipierro N, Paradiso A, De Gara L (2015). Involvement of DNA methylation in the control of cell growth during heat stress in tobacco BY-2 cells. Protoplasma.

[65] Qian Y, Hu W, Liao J, Zhang J, Ren Q (2019). The dynamics of DNA methylation in the maize (Zea mays L.) inbred line B73 response to heat stress at the seedling stage. Biochem Biophys Res Commun.

[66] He L, Huang H, Bradai M, Zhao C, You Y, Ma J (2022). DNA methylation-free Arabidopsis reveals crucial roles of DNA methylation in regulating gene expression and development. Nat Commun.

[67] Chatterjee R, Vinson C (2012). CpG methylation recruits sequence specific transcription factors essential for tissue specific gene expression. Biochim Biophys Acta - Gene Regul Mech.

[68] Costes E, Crespel L, Denoyes B, Morel P, Demene MN, Lauri PE et al. Bud structure, position and fate generate various branching patterns along shoots of closely related Rosaceae species: A review. Front Plant Sci. 2014;5(DEC). 10.3389/fpls.2014.00666 PMC4251308 25520729

[69] Tenreira T, Pimenta Lange MJ, Lange T, Bres C, Labadie M, Monfort A (2017). A specific gibberellin 20-oxidase dictates the flowering-runnering decision in diploid strawberry. Plant Cell.

[70] Yao N, Schmitz RJ, Johannes F (2021). Epimutations define a fast-ticking molecular clock in plants. Trends Genet.

[71] Herrera CM, Bazaga P, Pérez R, Alonso C (2021). Lifetime genealogical divergence within plants leads to epigenetic mosaicism in the shrub Lavandula latifolia (Lamiaceae). New Phytol.

[72] Molinier J, Ries G, Zipfel C, Hohn B (2006). Transgeneration memory of stress in plants. Nature.

[73] Sun RZ, Liu J, Wang YY, Deng X (2021). DNA methylation-mediated modulation of rapid desiccation tolerance acquisition and dehydration stress memory in the resurrection plant Boea hygrometrica. PLoS Genet.

[74] Murgia I, Giacometti S, Balestrazzi A, Paparella S, Pagliano C, Morandini P (2015). Analysis of the transgenerational iron deficiency stress memory in Arabidopsis thaliana plants. Front Plant Sci.

[75] Xu P, Chen H, Hu J, Cai W (2021). Potential evidence for transgenerational epigenetic memory in Arabidopsis thaliana following spaceflight. Commun Biol.

[76] Antro M, Van, Prelovsek S, Ivanovic S, Gawehns F, Niels CAM. DNA methylation in clonal Duckweed lineages (*Lemna minor L.*) reflects current and historical environmental exposures. 2022. 10.1111/mec.16757 PMC10100429 36324253

[77] Li J, Yang DL, Huang H, Zhang G, He L, Pang J (2020). Epigenetic memory marks determine epiallele stability at loci targeted by de novo DNA methylation. Nat Plants.

[78] Kollist H, Zandalinas SI, Sengupta S, Nuhkat M, Kangasjärvi J, Mittler R (2019). Rapid responses to abiotic stress: priming the Landscape for the Signal Transduction Network. Trends Plant Sci.

[79] Nievola CC, Carvalho CP, Carvalho V, Rodrigues E (2017). Rapid responses of plants to temperature changes. Temperature.

[80] Ohama N, Kusakabe K, Mizoi J, Zhao H, Kidokoro S, Koizumi S (2016). The transcriptional cascade in the heat stress response of arabidopsis is strictly regulated at the level of transcription factor expression. Plant Cell.

[81] Ohama N, Sato H, Shinozaki K, Yamaguchi-Shinozaki K (2017). Transcriptional Regulatory Network of Plant Heat Stress Response. Trends Plant Sci.

[82] Janni M, Gullì M, Maestri E, Marmiroli M, Valliyodan B, Nguyen HT (2020). Molecular and genetic bases of heat stress responses in crop plants and breeding for increased resilience and productivity. J Exp Bot.

[83] Huang Y, An J, Sircar S, Bergis C, Lopes CD, He X et al. HSFA1a modulates plant heat stress responses and alters the 3D chromatin organization of enhancer-promoter interactions. Nat Commun. 2023;14(1). 10.1038/s41467-023-36227-3 PMC9884265 36709329

[84] Sow MD, Le Gac AL, Fichot R, Lanciano S, Delaunay A, Le Jan I et al. RNAi suppression of DNA methylation affects the drought stress response and genome integrity in transgenic poplar. Vol. 232, New Phytologist. 2021. pp. 80–97. 10.1111/nph.17555 34128549

[85] Balasubramanian S, Sureshkumar S, Lempe J, Weigel D (2006). Potent induction of Arabidopsis thaliana flowering by elevated growth temperature. PLoS Genet.

[86] Suter L, Widmer A. Environmental heat and salt stress induce transgenerational phenotypic changes in *Arabidopsis thaliana*. PLoS ONE. 2013;8(4). 10.1371/journal.pone.0060364 PMC3621951 23585834

[87] Takeno K (2016). Stress-induced flowering: the third category of flowering response. J Exp Bot.

[88] Jagadish SVK, Way DA, Sharkey TD (2021). Plant heat stress: concepts directing future research. Plant Cell Environ.

[89] Kumar S, Gupta D, Nayyar H (2012). Comparative response of maize and rice genotypes to heat stress: Status of oxidative stress and antioxidants. Acta Physiol Plant.

[90] Lu C, Qiu N, Wang B, Zhang J (2003). Salinity treatment shows no effects on photosystem II photochemistry, but increases the resistance of photosystem II to heat stress in halophyte Suaeda salsa. J Exp Bot.

[91] Kadir S, Sidhu G, Al-Khatib K (2006). Strawberry (*Fragaria Xananassa Duch.*) Growth and productivity as affected by temperature. HortScience.

[92] Choi HG, Moon BY, Kang NJ. Correlation between strawberry (*Fragaria ananassa Duch*.) productivity and photosynthesis-related parameters under various growth conditions. Front Plant Sci. 2016;7(OCTOBER2016). 10.3389/fpls.2016.01607 PMC5080357 27833628

