# Supplementary material for: Epigenomic and transcriptomic persistence of heat stress memory in strawberry (Fragaria vesca)
Source: BMC Plant Biol. 2024 May 16;24:405. doi: 10.1186/s12870-024-05093-6 (PMC11096098; doi:10.1186/s12870-024-05093-6)
Supplement: Supplementary file 1 — Supplementary Material 1 [file 12870_2024_5093_MOESM1_ESM.pdf]

Supplementary figures

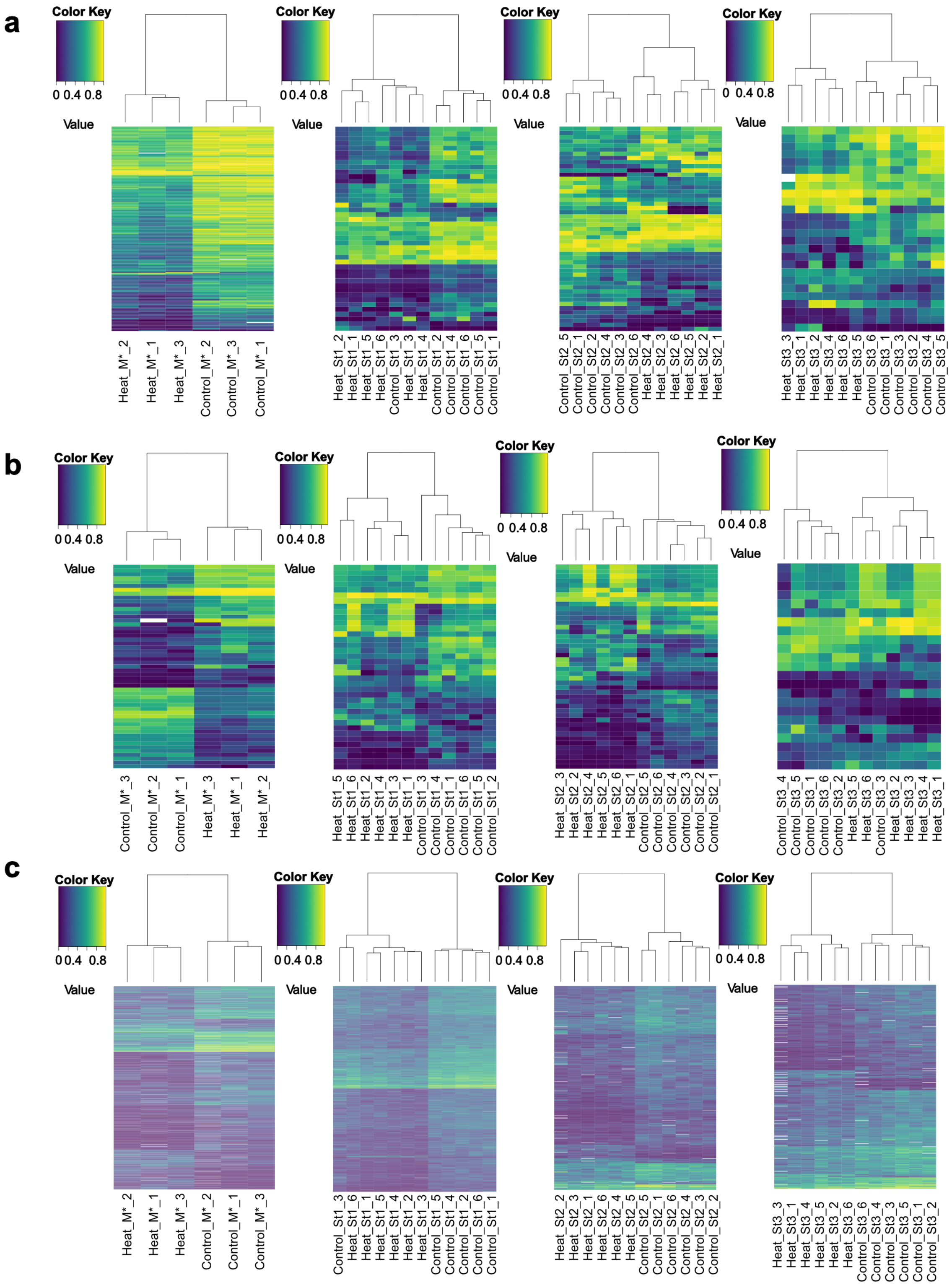

Fig. S1. Heatmap clustering groups based on significant DMRs (q < 0.05) in **a** CG, **b** CHG and **c** CHH. Methylome comparisons from control mother plants (CM*) against stress mother plants (HM*); control daughter plant (CSt1) vs. heat daughter plant (HSt1) from the first clonal propagation; control daughter plant (CSt2) vs. heat daughter plant (HSt2) from the second clonal propagation; control daughter plant (CSt3) vs. heat daughter plant (HSt3) from the third clonal propagation. *: samples were collected *in vitro*.

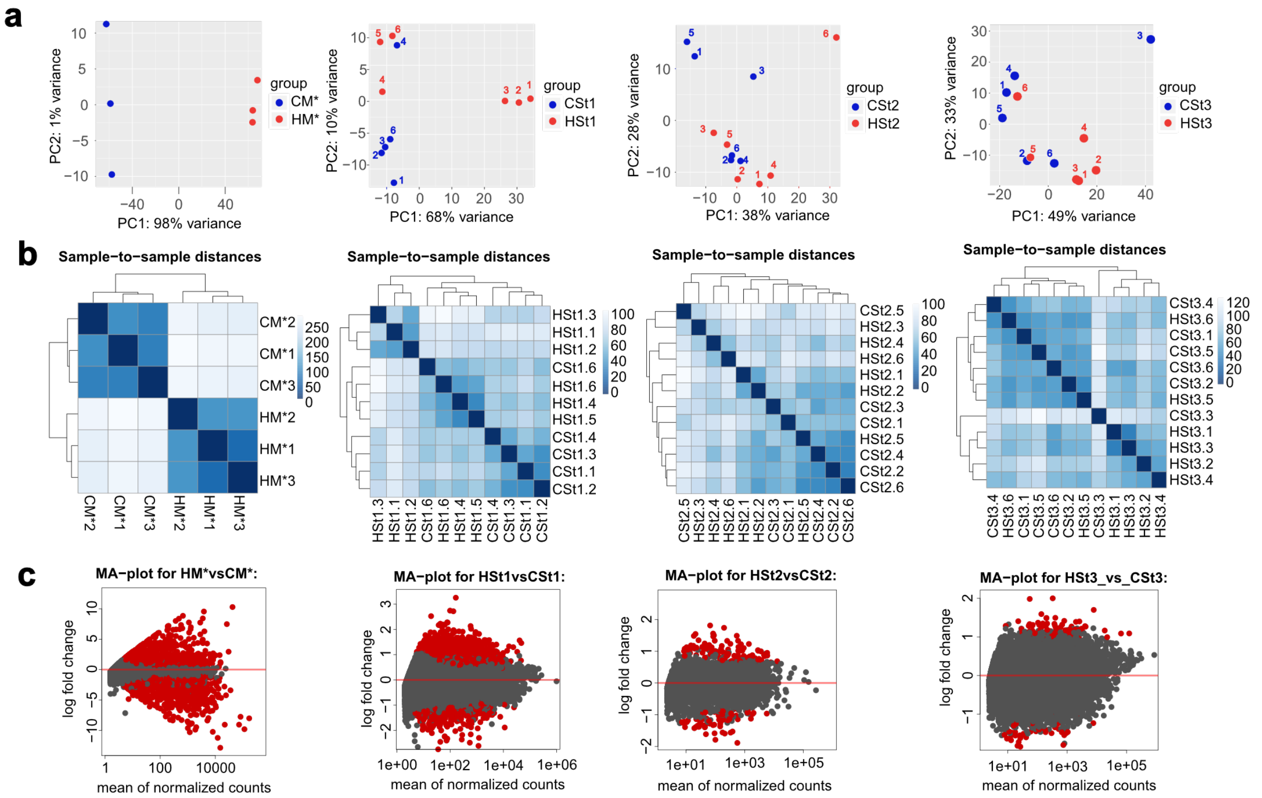

Fig. S2. RNA-seq analysis to detect differentially expressed genes (DEGs). a PCA plot showing the variability among control and heat stress samples. **b** Heatmap and cluster showing an overview of similarities and differences among samples. **c** Volcano plot showing the log2-fold change given to the variable mean of normalized counts. Red points indicate that the adjusted P value is less than 0.1. Plots were obtained by DESeq2 in the Galaxy platform. *: samples collected *in vitro*.

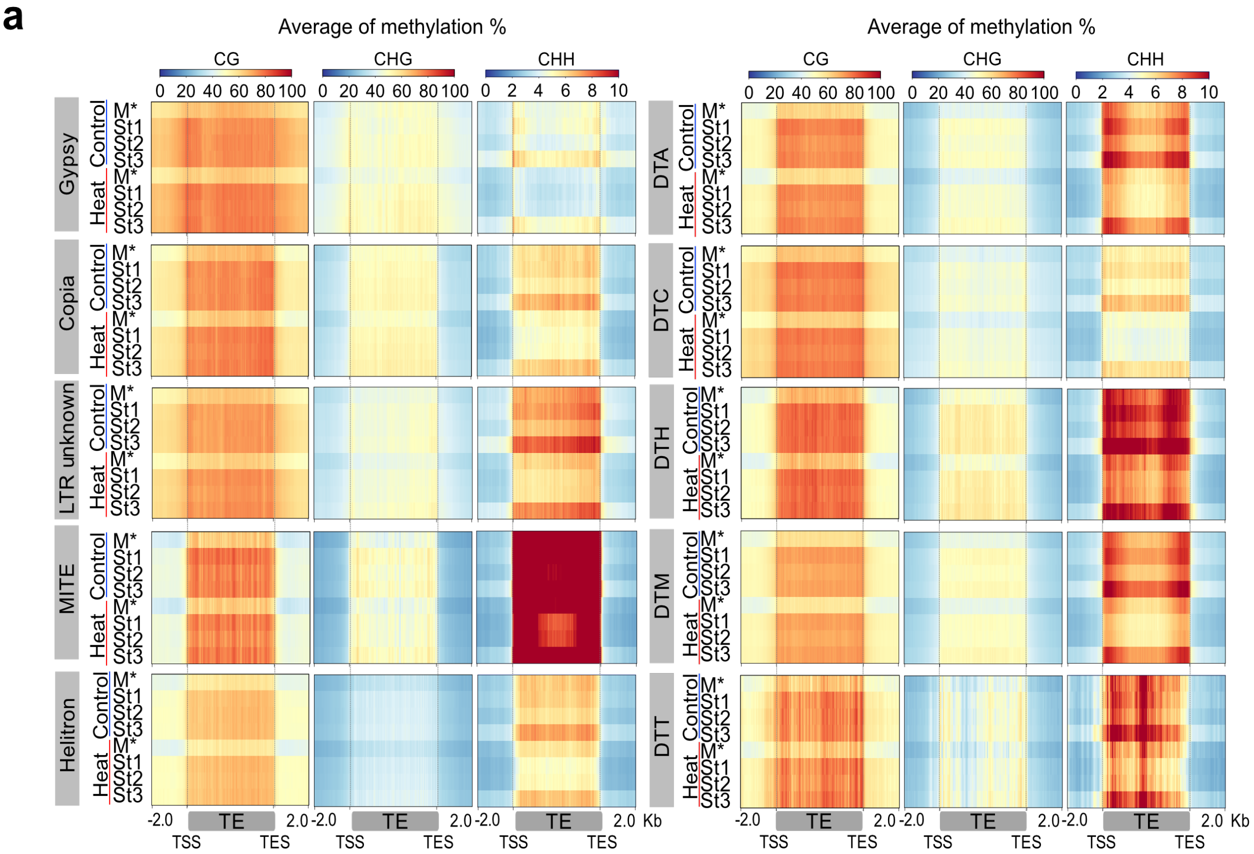

Fig. S3. Plot of global DNA methylation profiles of transposable elements (TE) families. Heatmaps showing DNA methylation profiles for all TE families separated by sequence context mCG (left), mCHG (center) and mCHH (right). The mean of the average DNA methylation percentage (within 50 bp sliding windows) was plotted for the TE bodies and 2 kb around the TSS and TES regions. Class I elements (retrotransposons): LTR-Copia, LTR-Gypsy. Class II elements (DNA transposons): TIR: Tc1-Mariner (DTT), hAT (DTA), Mutator (DTM), PIF-Harbinger (DTH), CACTA (DTC); Helitron; Miniature Inverted-Repeat Transposons (MITEs). M*: young mother plant (samples collected *in vitro*); St1: daughter plant of M; St2: daughter plant from St1; St3: daughter plant from St2. C: control, H: heat stress.

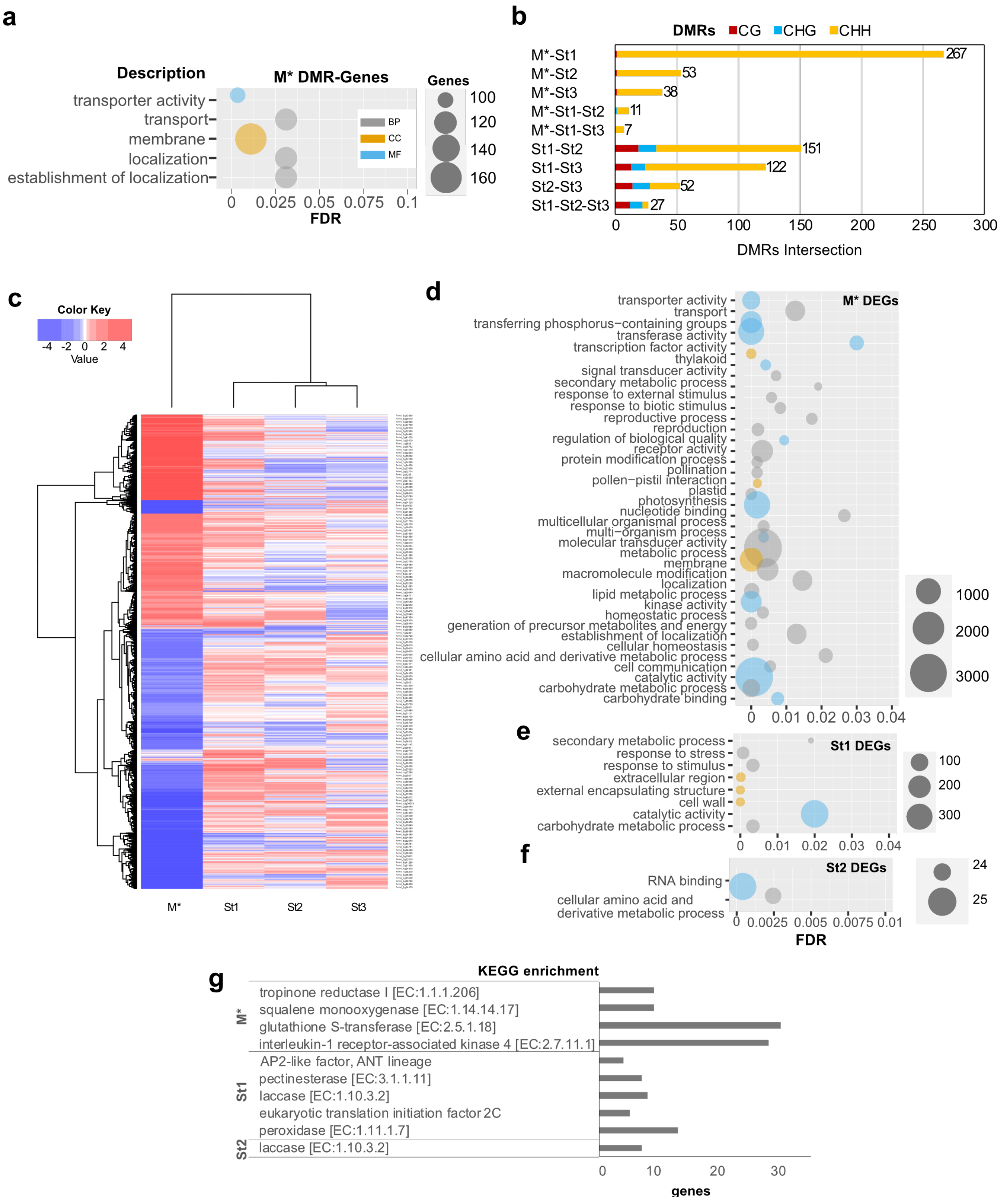

Fig. S4. Functional analysis of genes related to a DMR and differentially expressed genes (DEGs) in clonal and sexual generations. a Singular enrichment analysis (SEA) of the total number of genes related to a DMR (in the promoter and gene body); **b** Bar plot depicting counts of common DMR locations (minimum overlap: 1 bp) containing hypo- and hyperDMRs per context among all populations. Boxes above the plot indicate the color codes for DNA methylation: red for CG, blue for CHG and yellow for CHH sequence contexts. **c** Heatmap with DEGs present in at least one population (M*, St1, St2, St3). The upper cluster shows the link among samples from different clonal generations based on DEGs. **d** DEGs after heat stress in M*; **e** DEGs in St1; **f** DEGs in St2, performed using AgriGOv2 (P adj. <0.05). **g** KEGG enrichment analysis of the total number of DEGs from M*, St1 and St2 (results from clusterProfiler, P adj. <0.05). *: Samples collected *in vitro*.

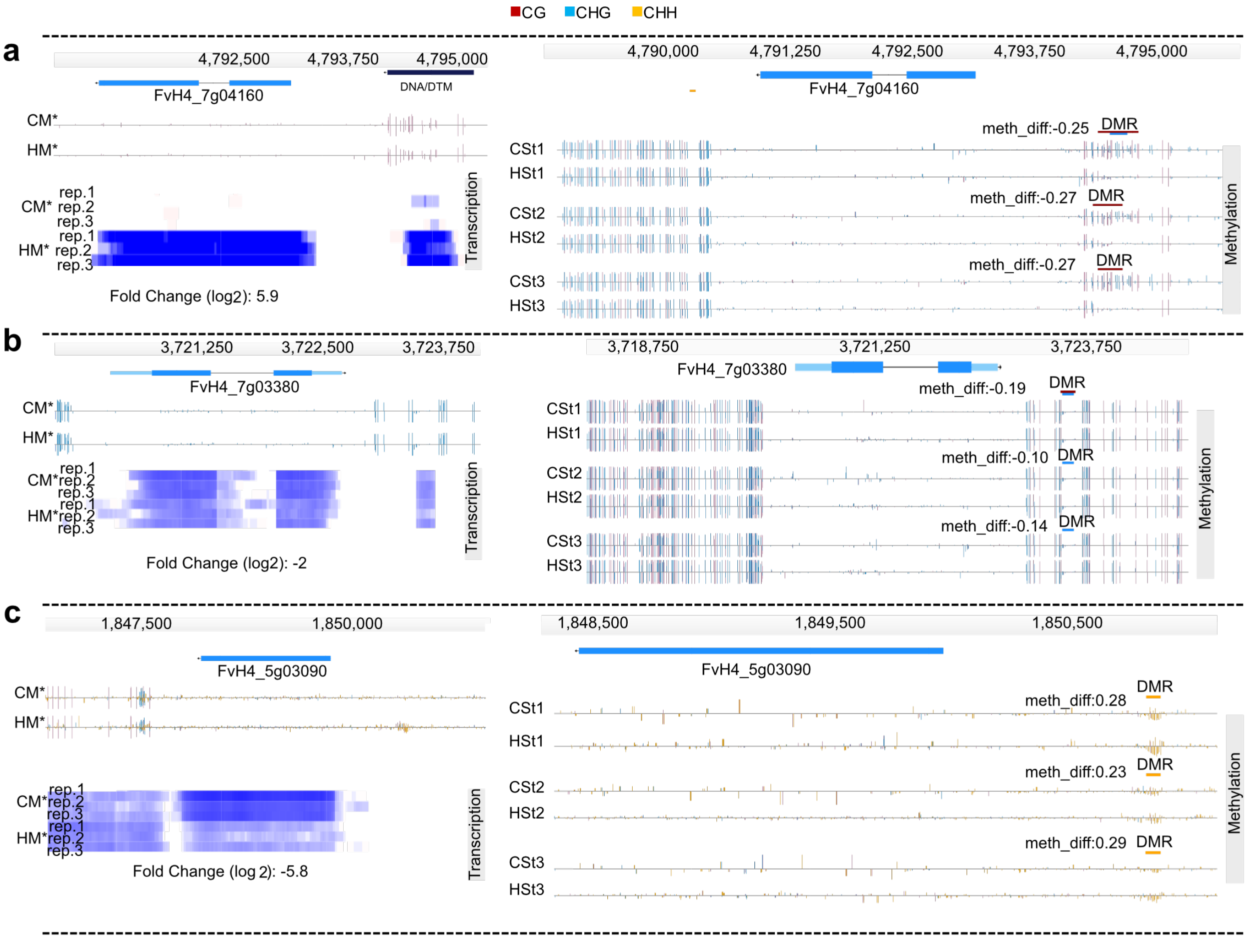

Fig. S5. Heat stress DMRs maintained over three asexual generations (St1, St2, St3) of genes that were differentially expressed in the mother plant (M*). Genome browser views of differentially expressed genes after heat stress in M* (left) and the DMRs located in promoter regions in St1, St2, and St3 at those same genes in the absence of stress (right). **a** PR5-like receptor kinase gene (FvH4_7g04160); **b** sequence-specific DNA binding transcription factor gene (FvH4_7g03380); **c** uncharacterized gene (FvH4_5g03090). Depicted are gene structures (top panels, UTRs in light blue, exons in blue), TEs (red and dark blue) and DNA methylation levels (histograms). Boxes above the histograms indicate identified DMRs with methylation difference ratios (color codes for DNA methylation: red for CG, blue for CHG and yellow for CHH contexts). *: Samples collected *in vitro*.

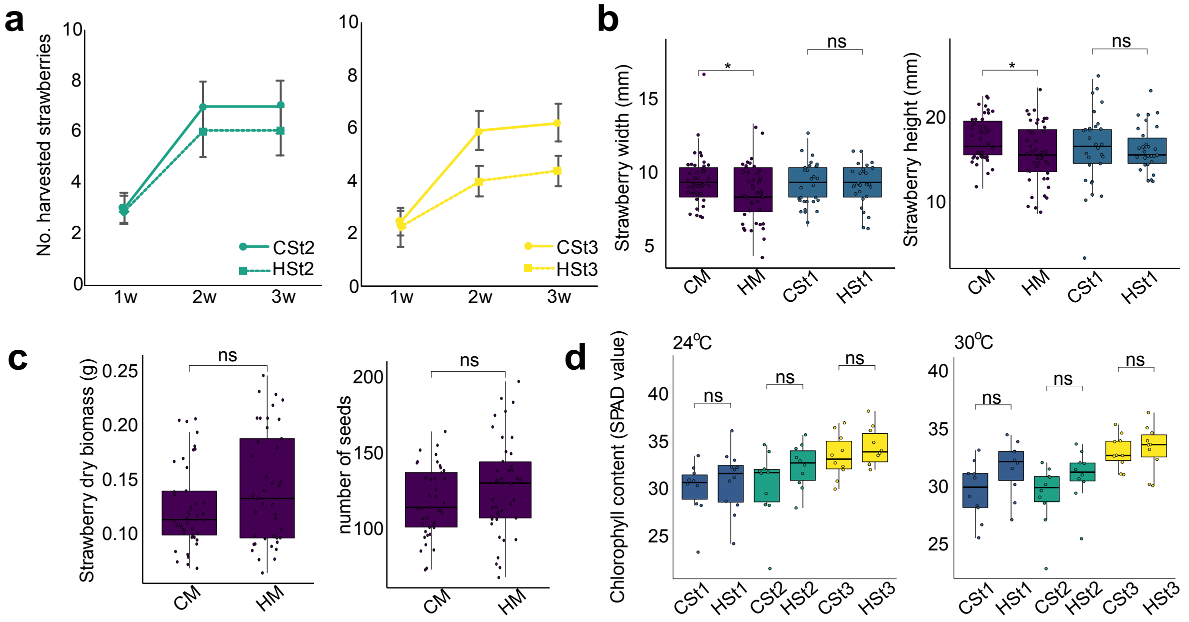

Fig. S6. Testing transgenerational inheritance of heat stress-induced phenotypic traits in *F. vesca*.

a Number of harvested strawberries during three consecutive weeks in the M and St generations. **b** Strawberry size: width and height (mm). **c** Strawberry dry biomass and number of seeds per fruit. **d** Total leaf chlorophyll content by the measurement of the Soil Pant Analysis Development (SPAD) values by chlorophyll meter of plants submitted to an ascending temperature gradient (+5°C every 24 h). All statistical analysis comparisons applied with Wilcoxon rank sum tests: *P ≤ 0.05; ns: not significant. HM: heat-stressed mother plant; CM: control mother plant; St1: first daughter plant from M; St2: daughter plant from St1; St3: daughter plant from St2.
